# Comparative Genomic Analysis Reveals Genetic Diversity and Pathogenic Potential of Haemophilus seminalis and Emended Description of Haemophilus seminalis

**DOI:** 10.1128/spectrum.04772-22

**Published:** 2023-06-29

**Authors:** Xiaowei Chen, Hanyun Zhang, Junhui Feng, Lei Zhang, Minling Zheng, Haimin Luo, Huiyan Zhuo, Ning Xu, Xuan Zhang, Cha Chen, Pinghua Qu, Youqiang Li

**Affiliations:** a The Second Clinical College of Guangzhou University of Chinese Medicine, Guangzhou, China; b Department of Laboratory Medicine, The Affiliated Hexian Memorial Hospital of Southern Medical University, Guangzhou, China; c Guangzhou Kingmed Center for Clinical Laboratory, Guangzhou, China; d Department of Clinical Laboratory, Guangdong Women and Children Hospital, Guangzhou, China; e Department of Clinical Laboratory, The Second Affiliated Hospital of Guangzhou University of Chinese Medicine, Guangdong Provincial Hospital of Chinese Medicine, Guangzhou, China; Indian Institute of Technology Hyderabad

**Keywords:** *Haemophilus seminalis*, comparative genomics, genetic diversity, phylogeny, taxonomy

## Abstract

Haemophilus seminalis is a newly proposed species that is phylogenetically related to Haemophilus haemolyticus. The distribution of *H. seminalis* in the human population, its genomic diversity, and its pathogenic potential are still unclear. This study reports the finding of our comparative genomic analyses of four newly isolated Haemophilus strains (SZY H8, SZY H35, SZY H36, and SZY H68) from human sputum specimens (Guangzhou, China) along with the publicly available genomes of other phylogenetically related Haemophilus species. Based on pairwise comparisons of the 16S rRNA gene sequences, the four isolates showed <98.65% sequence identity to the type strains of all known Haemophilus species but were identified as belonging to *H. seminalis*, based on comparable phenotypic and genotypic features. Additionally, the four isolates showed high genome-genome relatedness indices (>95% ANI values) with 17 strains that were previously identified as either “*Haemophilus intermedius*” or hemin (X-factor)-independent H. haemolyticus and therefore required a more detailed classification study. Phylogenetically, these isolates, along with the two previously described *H. seminalis* isolates (a total of 23 isolates), shared a highly homologous lineage that is distinct from the clades of the main H. haemolyticus and Haemophilus influenzae strains. These isolates present an open pangenome with multiple virulence genes. Notably, all 23 isolates have a functional heme biosynthesis pathway that is similar to that of *Haemophilus parainfluenzae*. The phenotype of hemin (X-factor) independence and the analysis of the *ispD*, *pepG*, and *moeA* genes can be used to distinguish these isolates from H. haemolyticus and H. influenzae. Based on the above findings, we propose a reclassification for all “*H*. *intermedius*” and two H. haemolyticus isolates belonging to *H. seminalis* with an emended description of *H. seminalis.* This study provides a more accurate identification of Haemophilus isolates for use in the clinical laboratory and a better understanding of the clinical significance and genetic diversity in human environments.

**IMPORTANCE** As a versatile opportunistic pathogen, the accurate identification of Haemophilus species is a challenge in clinical practice. In this study, we characterized the phenotypic and genotypic features of four *H. seminalis* strains that were isolated from human sputum specimens and propose the “*H*. *intermedius*” and hemin (X-factor)-independent H. haemolyticus isolates as belonging to *H. seminalis*. The prediction of virulence-related genes indicates that *H. seminalis* isolates carry several virulence genes that are likely to play an important role in its pathogenicity. In addition, we depict that the genes *ispD*, *pepG*, and *moeA* can be used as biomarkers for distinguishing *H. seminalis* from H. haemolyticus and H. influenzae. Our findings provide some insights into the identification, epidemiology, genetic diversity, pathogenic potential, and antimicrobial resistance of the newly proposed *H*. *seminalis*.

## INTRODUCTION

The genus Haemophilus, which is classified in the family *Pasteurellaceae* of the order *Pasteurellales*, consists of pleomorphic Gram-negative coccobacilli with growth requirements for X-factors and V-factors (1). They are part of the human commensal flora and are most commonly found in the upper respiratory tract, oral cavity, and mucous membranes (1–11). So far, 13 Haemophilus species have been isolated from human sources, of which the names of 11 species are recognized by the International Committee of Systematics of Prokaryotes (ICSP) as validly published (H. influenzae, H. aegyptius, H. ducreyi, H. haemolyticus, H. pittmaniae, H. parahaemolyticus, H. parainfluenzae, H. paraphrohaemolyticus, H. sputorum, H. massiliensis, and H. seminalis) (1–11). The names of two species, “H. quentini” and “H. intermedius”, need to be validated to be recognized by ICSP (1–11).

H. influenzae is a clinically relevant bacterial pathogen that can cause respiratory infections and invasive diseases (1, 2). In contrast, H. haemolyticus is a part of the commensal flora in the human respiratory tract that can cause invasive infections as an opportunistic pathogen (3–6). For a proper understanding of the etiology of a disease, the accurate identification of the pathogen is necessary. This becomes problematic in the case of Haemophilus species, as the currently practiced phenotypic methods often result in species misidentification due to their limited differentiating characteristics (6, 11–14). For instance, the H. haemolyticus, “*H*. *intermedius*,” and “*H. quentini*” are frequently misidentified as nontypeable H. influenzae (NTHi) (11–14). Even the identification accuracy of molecular methods is compromised due to the extensive recombination events and horizontal gene transfer among the related Haemophilus members (12, 13). It is reported that the false identification of Haemophilus spp. in some clinical microbiology laboratories is as high as 40% (6).

*H. seminalis*, which is a newly proposed species that is phylogenetically related to H. haemolyticus, “*H*. *intermedius*”, and “*H*. *quentini*”, was first isolated from clinical semen samples as a suspected case of Neisseria gonorrhoeae infection by using chocolate agar PolyViteX (9). So far, there are no other reports of *H. seminalis* identification from other human sources. As Haemophilus was reported as an etiological agent of nongonococcal urethritis that was associated with oral sex (15), we suspected the possibility of the clinical isolates (identified as *H. seminalis*) being an opportunistic pathogen causing urinary and genital tract infections.

In this study, we provided the polyphasic characterization of four strains (SZY H8, SZY H35, SZY H36, and SZY H68) isolated from human sputum in Guangzhou, China. Additionally, a comparative genomic analysis of the four isolates as well as the publicly available genomes of others that are phylogenetically related to Haemophilus species was performed. Finally, we performed an emended description for *H*. *seminalis.* Overall, this study provides some insights into the identification, epidemiology, genetic diversity, pathogenic potential, and antimicrobial resistance of the newly proposed *H*. *seminalis*.

## RESULTS

### Clinical information of *H. seminalis* strains.

**FIG 1:**
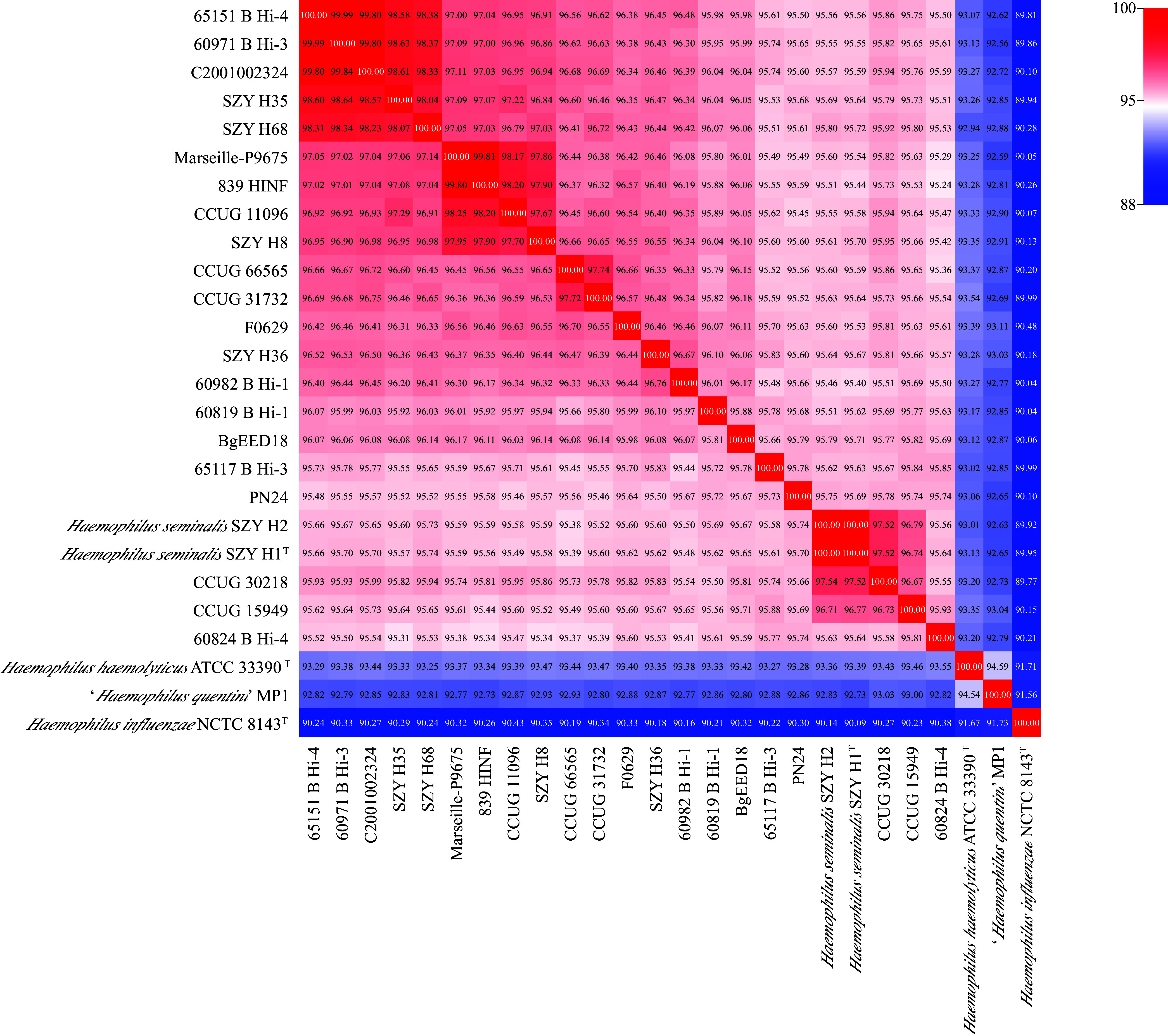
Heat map of the average nucleotide identity (ANI) of 26 Haemophilus species strains. The percentage identity among strains is shown.

Four isolates, designated strains SZY H8, SZY H35, SZY H36, and SZY H68, were obtained from clinical sputum specimens of four hospitalized patients (Table S1). The sequences of all other genomes related to Haemophilus species used in the current study were retrieved from the NCBI GenBank database (Table S2). Our four isolates exhibited DNA-DNA relatedness values of above 95% average nucleotide identity (ANI) values with the two isolates SZY H1^T^ and SZY H2 (previously identified as *H. seminalis*), 14 “*H. intermedius*” or hi*Hh* isolates, two H. haemolyticus isolates (strains BgEED18 and C2001002324), and one genome labeled as Haemophilus species in the GenBank database (Table 1; Fig. 1). The clinical sources of the above isolates are human semen, sputum, bronchoalveolar lavage fluid, urine, eye, cerebrospinal fluid, feces, ascitic fluid, and pleural fluid (Table 1), and their reports originate from China, Bangladesh, Denmark, the USA, Sweden, Australia, and France.

**TABLE 1:** *H*. *seminalis* strains used in this study

Strain	Anatomical site	Country of origin	Yr of isolation	Genome size (bp)	Contigs	GC content (%)	Accession no.	Original species designation	Reference
SZY H1^T^	Semen	China	2018	2,061,978	21	38.20%	VCED00000000	*H. seminalis*	9
SZY H2	semen	China	2019	2,059,120	21	38.20%	VXDF00000000	*H. seminalis*	9
SZY H68	Sputum	China	2018	1,902,918	1	38.40%	CP091469	-	This work
SZY H8	Sputum	China	2019	1,855,861	15	38.10%	JAHKJY000000000	-	This work
SZY H35	Sputum	China	2018	1,915,946	12	38.10%	JAHKJZ000000000	-	This work
SZY H36	Sputum	China	2018	1,861,813	10	38.20%	JAHKKA000000000	-	This work
BgEED18	Feces	Bangladesh	2020	1,772,070	27	38.30%	CABFLR00000000	H. haemolyticus	44
PN24	Urine	Denmark	2005	1,938,749	67	38.10%	SDPK00000000	H. haemolyticus	14
C2001002324	Sputum	USA	2001	1,882,180	20	38.20%	QEPL00000000	H. haemolyticus	45
CCUG 31732	Ascitic fluid	Sweden	1993	1,892,724	80	38.40%	SDPJ00000000	H. haemolyticus	14
CCUG 11096	Pleural fluid	Sweden	1981	1,934,421	60	38.20%	SDPG00000000	H. haemolyticus	14
CCUG 15949	Eye	Sweden	1984	1,991,233	43	38.10%	SDPH00000000	H. haemolyticus	14
CCUG 30218	Cerebrospinal fluid	Sweden	1992	1,986,818	66	38.20%	SDPI00000000	H. haemolyticus	14
CCUG 66565	Sputum	Sweden	2014	1,926,945	18	38.30%	LZOX00000000	H. haemolyticus	11
65117 B Hi-3	Bronchoalveolar lavage	Australia	2011	1,929,375	16	38.20%	SDPE00000000	H. haemolyticus	42
60824 B Hi-4	Bronchoalveolar lavage	Australia	2010	1,980,986	25	38.30%	SDPB00000000	H. haemolyticus	42
60819 B Hi-1	Bronchoalveolar lavage	Australia	2010	1,915,804	13	38.30%	SDPA00000000	H. haemolyticus	42
60982 B Hi-1	Bronchoalveolar lavage	Australia	2012	1,921,666	13	38.30%	SDPD00000000	H. haemolyticus	42
65151 B Hi-4	Bronchoalveolar lavage	Australia	2011	1,897,573	13	38.20%	SDPF00000000	H. haemolyticus	42
60971 B Hi-3	Bronchoalveolar lavage	Australia	2012	1,895,161	13	38.20%	SDPC00000000	H. haemolyticus	42
839 HINF	Bronchoalveolar lavage	USA	2013	1,937,310	47	38.20%	JURC00000000	H. influenzae	43
F0629	Oral cavity	USA	2015	1,805,716	1	38.40%	CP027235	H. haemolyticus	11
Marseille-P9675	sputum	France	2021	1,927,163	19	38.20%	CAKASP010000000	Haemophilus sp.	-

### Phenotypic features.

The four isolates SZY H8, SZY H35, SZY H36, and SZY H68 were observed to be Gram-negative, nonacid-fast, and nonmotile. The cells were pleomorphic rods or coccobacilli without any filaments or capsules (Fig. S1). Unlike the type strain SZY H1^T^ that grows at 28 to 40°C, the four isolates can grow at 42°C. Under conditions of 35°C and 5% CO_2_, they do not exhibit growth on Columbia blood agar, MacConkey agar, brain heart infusion (BHI) agar, or Mueller-Hinton (MH) agar but grow well on Haemophilus chocolate 2 agar, chocolate agar PolyViteX. In addition, satellite growth was observed around the discs containing V-factors or V+X-factors on BHI agar and a streak of Staphylococcus aureus ATCC 25923 on Columbia blood agar. However, only isolate SZY H68 was found positive for hemolytic activity.

The four isolates were positive for glucose and sucrose fermentation tests but were negative for maltose fermentation, esculin hydrolysis, Voges-Proskauer, Simmons’s citrate, and motility tests. They show positive results in catalase, urease, alkaline phosphatase, leucine arylamidase, acid phosphatase, and naphthol-AS-BI-phosphohydrolase activity test but not for arginine dihydrolase, gelatinase, esculin, lysine decarboxylase activities, H_2_S, esterase (C4), esterase lipase (C8), lipase (C14), valine arylamidase, cystine arylamidase, trypsin arylamidase, α-chymotrypsin, α-galactosidase, β-galactosidase, β-glucuronidase, α-glucosidase, β-glucosidase, N-acetyl-β-glucosaminase, α-mannosidase, β-fucosidase, proline arylaminase, or penicillinase activities. Combining our results and that obtained from the data of Culture Collection University of Gothenburg (https://www.ccug.se/), the four isolates showed variable test results for oxidase activity, hemolytic activity, nitrite reduction test, indole production test, and γ-glutamyl transferase activity. Other physiological and biochemical characteristics of the four isolates and their related strains are summarized in Table 2.

**TABLE 2:** Different phenotypic characteristics among *H. seminalis* strains and related type strains^*a*^

Characteristic	1	2	3	4	5	6	7^*b*^	8^*b*^	9^*b*^	10^*b*^	11	12
Growth temp range (°C)	28 to 40	28 to 40	28–42	28–42	28–42	28–42	ND	ND	ND	ND	28 to 42	28 to 37
Optimum growth temp (°C)	32 to 37	32 to 37	35 to 37	35 to 37	35 to 37	35 to 37	ND	ND	ND	ND	37	35
Hemolysis	−	−	−	+	−	−	ND	ND	ND	ND	+	−
X-factor requirement	−	−	−	−	−	−	ND	ND	ND	ND	+	+
Reduction of nitrate	+	+	+	+	+	+	−	−	−	−	+	W
Urease	+	+	+	+	W	+	+	+	+	+	+	+
Indole production	−	−	−	−	−	−	+	−	−	−	−	+
Ornithine decarboxylase	−	−	−	−	−	−	+	+	+	+	−	+
Acid from fructose	−	−	−	−	−	−	ND	−	−	ND	+	−
Acid from maltose	−	−	−	−	−	−	ND	+	+	ND	+	−
Acid from sucrose	+	+	+	+	+	+	ND	−	−	ND	−	−

a The strains are indicated as: 1, SZY H1^T^; 2, SZY H2; 3, SZY H68; 4, SZY H8; 5, SZY H35; 6, SZY H36; 7, CCUG 15949; 8; CCUG 31732; 9, CCUG 11096; 10, CCUG 30218; 11, H. haemolyticus NCTC 10659^T^; and 12, H. influenzae ATCC 10211. +, positive; –, negative; W, weak positive reaction; ND, not detected.

b The data were taken from the Culture Collection University of Gothenburg (https://www.CCUG.se).

The major fatty acids (>10%) of strains SZY H1^T^, SZY H2, SZY H68, SZY H8, SZY H35, and SZY H36 were identified as C_14:0_, C_16:0_, and summed feature 3 (C_16:1_*_ω_*_7_*_c_* and/or C_16:1_ *_ω_*_6_*_c_*), which were similar to those of H. haemolyticus NCTC 10659^T^ and H. influenzae ATCC 10211 (Table 3).

**TABLE 3:** Cellular fatty acid compositions of *H*. *seminalis* strains and related type strains^*a*^

Fatty acid	1	2	3	4	5	6	7^*b*^	8	9
Saturated fatty acids									
C_14:0_	19.34	16.40	20.57	19.99	19.48	20.14	27.90	15.16	18.04
C_16:0_	33.70	33.70	35.99	31.38	34.25	31.39	24.50	28.66	32.49
C_18:0_	1.94	3.60	2.04	1.31	1.85	1.53	0.80	5.91	Tr
C_20:0_	Tr	0.60	Tr	–	Tr	Tr	–	1.50	–
C_18:1_ ω9*c*	1.41	–	1.56	1.10	1.97	1.30	3.90	1.39	Tr
C_20:1_ ω7*c*	Tr	0.70	Tr	Tr	–	Tr	–	1.12	–
Unsaturated fatty acids									
Summed Feature 2	9.87	10.80	9.04	11.46	9.44	10.06	9.40	12.32	8.55
Summed Feature 3	28.14	27.70	25.50	29.97	27.61	30.38	30.20	28.08	37.31
Summed Feature 5	1.39	1.20	1.38	1.11	1.65	1.21	0.80	1.46	Tr
Summed Feature 8	2.17	1.60	1.78	1.86	1.90	1.76	–	2.37	1.16

a The strains are indicated as: 1, SZY H1^T^; 2, SZY H2; 3, SZY H68; 4, SZY H8; 5, SZY H35; 6, SZY H36; 7, CCUG 15949; 8, H. haemolyticus NCTC 10659^T^; and 9, H. influenzae ATCC 10211. All of the data are from this study. The values are the percentages of total fatty acids. Tr, trace (<1%); –, not detected. Summed feature 2 contained iso-C_16:1_ and/or C_14:0_ 3-OH. Summed feature 3 contained C_16:1_ ω7*c* and/or C_16:1_ ω6*c*. Summed feature 5 contained C_18:2_ ω6,9*c* and/or C_18:0_ ante. Summed feature 8 contained C_18:1_ ω7*c* and/or C_18:1_ ω6*c*.

b The data were taken from the Culture Collection University of Gothenburg (https://www.CCUG.se).

### Genomic and phylogenomic analyses.

**FIG 2:**
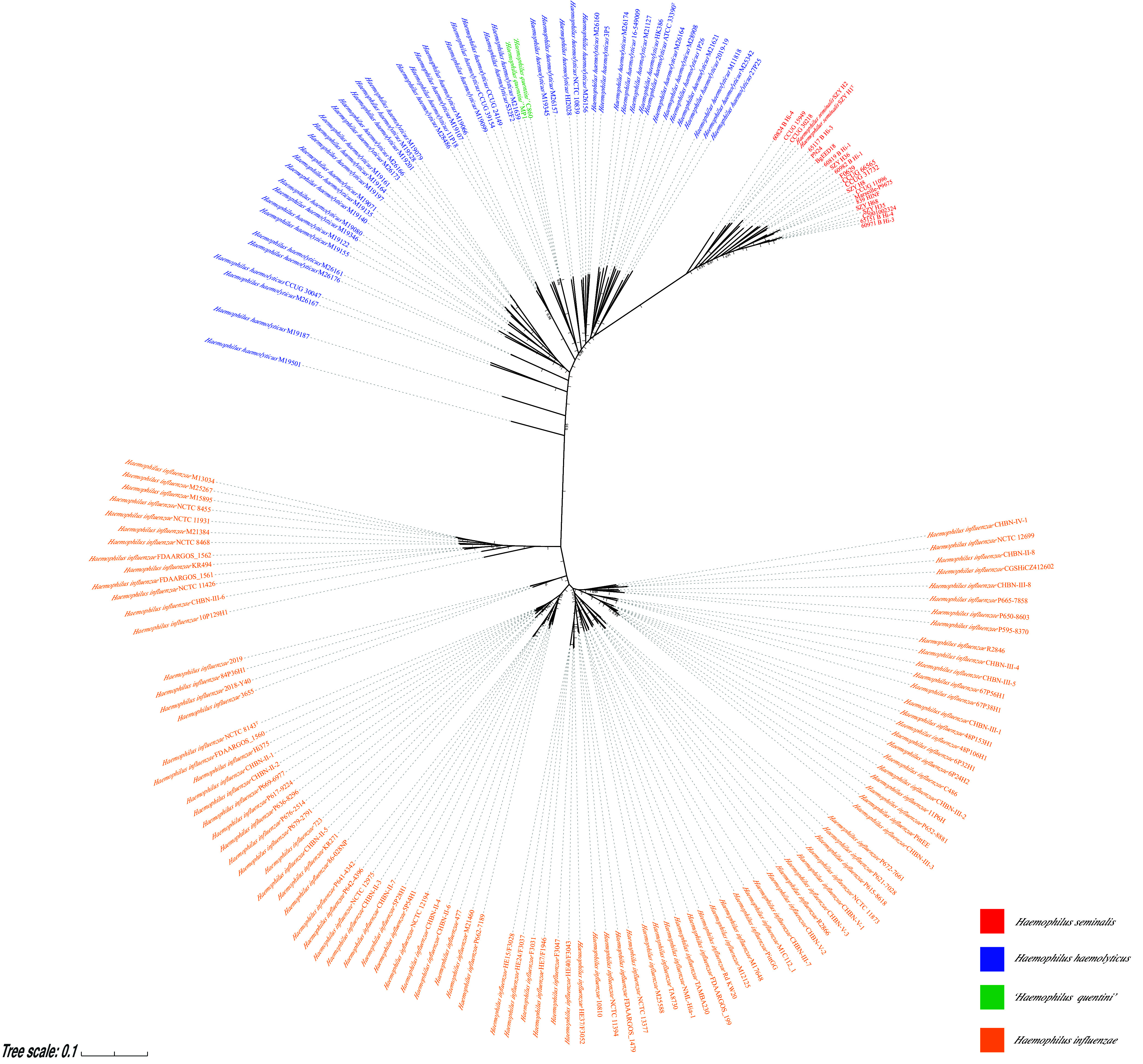
A maximum likelihood phylogenomic tree was constructed based on 621 core genes for 173 Haemophilus spp. genomes. The different colors of strain IDs stand for different species, as defined in the legend. The red strain ID represents *H. seminalis*, the blue strain ID represents H. haemolyticus, the green strain ID represents “*H. quentini*”, and the orange strain ID represents H. influenzae. The numbers at the nodes are support values that were estimated with 1,000 bootstrap replicates. Only bootstrap values of ≥0.9 are indicated.

Phylogenomic reconstruction of the 173 Haemophilus genomes based on core genes (Data Set S1) was carried out to determine the relationships between the *H. seminalis*, H. haemolyticus, “*H*. *intermedius*”, “*H*. *quentini*”, and H. influenzae isolates. The 2 *H*. *seminalis* strain isolates (strains SZY H1^T^ and SZY H2), the isolates of this study (strains SZY H68, SZY H8, SZY H35, and SZY H36), 14 previously described “*H*. *intermedius*” (hi*Hh*) isolates, and strains BgEED18 and C2001002324 identified as H. haemolyticus, and 1 Haemophilus sp. strain Marseille-P9675 (Table 1) shared a highly homologous lineage, which is distinct from the main H. haemolyticus and H. influenzae clades (Fig. 2). These 23 isolates demonstrated DNA-DNA relatedness values of 95.24 to 100% ANI and 63.00 to 100% digital DNA-DNA hybridization (dDDH) to each other (Table S3). They had the next highest genome relatedness indices to the isolates belonging to the main H. haemolyticus clade, 92.16 to 93.83% ANI values (Fig. S2).

The genome size of the 23 isolates ranges from 1.78 Mb to 2.06 Mb, with GC contents of 38.1 to 38.4 mol%. The complete genome of strain SZY H68 was circular with a genome size of 1,902,920 bp. In total, 1,828 predicted genes were annotated, including 1,731 protein-coding genes, 81 encoded RNAs (58 tRNA genes, 4 ncRNA genes, 7 copies of 5S rRNA genes, 6 copies of 16S rRNA genes, and 6 copies of 23S rRNA genes), and 16 pseudogenes. No plasmids were detected in the genome of strain SZY H68.

### rRNA and protein-encoding genes analysis.

In total 166 sequences of 16S rRNA genes (996 bp) were retrieved from these 173 Haemophilus spp. genomes. These include 20 *H*. *seminalis*, 46 H. haemolyticus, 2 “*H. quentini*” and 98 H. influenzae (5 H. influenzae type a (Hia), 5 H. influenzae type b (Hib), 2 H. influenzae type c (Hic), 4 H. influenzae type e (Hie), 4 H. influenzae type f (Hif), and 78 nontypeable H. influenzae (NTHi)). A phylogenetic tree based on the 16S rRNA gene showed that the clade containing the *H*. *seminalis*, H. haemolyticus, and “*H. quentini*” isolates are distinct from the H. influenzae clade. However, the 20 *H*. *seminalis* isolates were not clustered together but rather were distributed into several heterogenous subclades within the H. haemolyticus clade (Fig. S3A). The isolates SZY H8, SZY H35, SZY H36, SZY H68, C2001002324, 60971 B Hi-3, 65151 B Hi-4, 839 HINF, 60819 B Hi-1, 65117 B Hi-3, and 60824 B Hi-4 clustered together within one subclade, and they have 99.03 to 100% 16S rRNA sequence identities between them. The sequence identities of these 11 isolates are 97.99 to 98.49% with H. haemolyticus CCUG 12834^T^ and 97.25 to 97.74% similarities with *H*. *seminalis* SZY H1^T^. Considering the low sequence identities (below the 98.86% cutoff value for species delineation), the four isolates obtained in this study (SZY H8, SZY H35, SZY H36, SZY H68) were considered to be a putative novel species of the genus Haemophilus. For further comparative studies, isolate SZY H68 was selected as the representative strain. The six copies of 16S rRNA genes extracted from the genome (accession number: CP091469) also showed identical sequences with considerable divergence (<98.65% similarities) to the type strains of valid-published species. Further, the two *H*. *seminalis* strains, SZY H1^T^ and SZY H2, and the H. haemolyticus strain CCUG 15949 formed a single subclade, with 99.78 to 100% sequence similarity within themselves (Table S4).

The phylogenetic analysis for these Haemophilus isolates was based on the 23S rRNA gene and the previously described single-copy housekeeping genes such as *rpoB*, *recA*, *frdB*, *mdh*, *adk*, *atpG*, and *pgi* (16). The basic topology of the phylogenetic tree created on the basis of these genes also showed that all the *H*. *seminalis*, H. haemolyticus, and “*H. quentini*” isolates were clustered together with a big clade, which was quite similar to that of the 16S rRNA gene. Therefore, the single gene analyses of 16S rRNA, 23S rRNA, and the above housekeeping genes were unable to distinguish *H. seminalis* from H. haemolyticus and “*H. quentini*” (Fig. S3).

**FIG 3:**
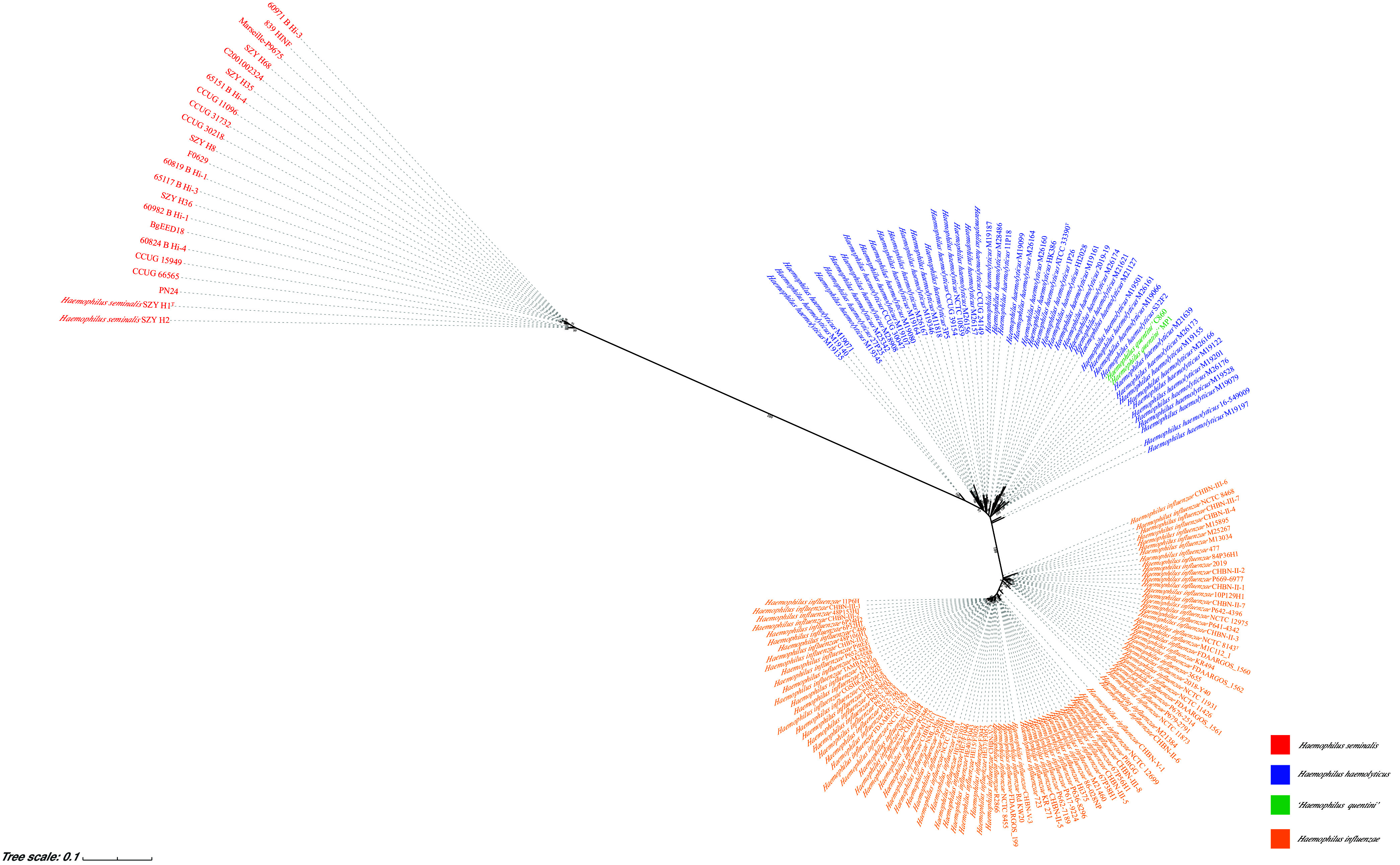
Maximum likelihood tree, based on a partial *ispD* gene (about 677 bp), showing the phylogenetic relationship of 173 Haemophilus spp. strains. The gene sequences were aligned using MUSCLE. The tree was made using IQ-TREE. The different colors of strain IDs stand for different species, as defined in the legend. The red strain ID represents *H. seminalis*, the blue strain ID represents H. haemolyticus, the green strain ID represents “*H. quentini*”, and the orange strain ID represents H. influenzae. The numbers at the nodes are support values that were estimated with 1,000 bootstrap replicates. Only bootstrap values of ≥90 are indicated.

A further search for effective marker genes was also performed based on pan/core genome analysis. The genes *ispD*, *pepG*, and *moeA* retrieved from these 173 Haemophilus spp. genomes were found to be conserved and were selected for comparative analysis. Comparison of *ispD* gene sequences showed that 23 isolates clustered together and had a 97.35 to 100% sequence similarity, which is distinct from H. haemolyticus and H. influenzae clades (Fig. 3). They shared 77.60% to 78.01% similarities with H. haemolyticus ATCC 33390^T^ and 76.39% to 76.95% similarities with H. influenzae NCTC 8143^T^ (Table S5). We also performed a phylogenetic analysis for these Haemophilus isolates based on the *pepG* and *moeA* genes. The dendrograms revealed that the phylogenetic positions of the isolates were similar to that of the *ispD* gene (Fig. S4). However, the resolving power of the *ispD* gene was found to be relatively better than those of the *pepG* and *moeA* genes.

### Pangenome analyses.

**FIG 4:**
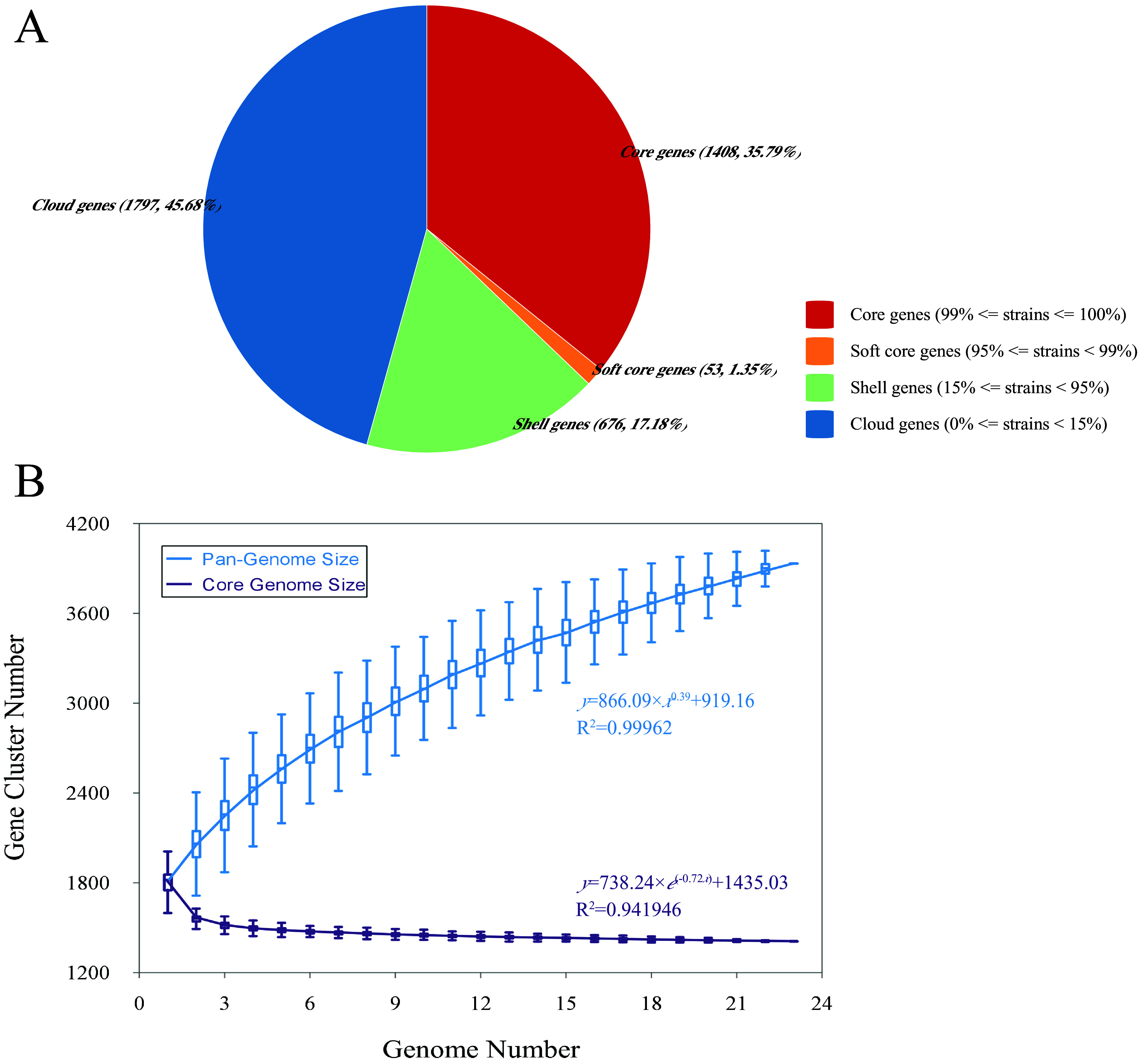
Pangenome analysis of *H. seminalis* species. (A) Pie chart indicating the gene set in the pangenome. (B) Increase and decrease in gene families in the pangenome (blue) and the core genome (violet), respectively. Gene accumulation curves were plotted as a function of the number of genomes that were sequentially added (*n* = 23) in the power law regression model. The trajectory of the pangenome shows that the pangenome is open.

The pangenome of all 23 *H. seminalis* strains comprised 3,934 genes, including 1,408 core genes (present in 99 to 100% of genomes), 53 softcore genes (present in 95 to 99% of genomes), 676 shell genes (present in 15 to 95% of genomes), and 1,797 cloud genes (present in 0 to 15% of genomes) (Fig. 4A). For the core genes, 84.7% (1,193/1,408) encoded a known function, while only 15.3% (215/1,408) were assigned to hypothetical proteins. The accessory genome comprised 676 shell (17.2%) and 1,797 cloud (45.7%) genes. Remarkably, 76.5% (1,892/2,473) of the accessory genes were considered to code for hypothetical proteins, and 54.3% of the predicted genes (2,137/3,934) have no known function (Data Set S1).

The clusters of orthologous groups (COG) analysis showed that the core genome proteins were enriched in translation, ribosomal structure and biogenesis (J), energy production and conversion (C), amino acid transport and metabolism (E), and inorganic ion transport and metabolism (P) genes. This indicates that these genes were more critical to the survival of *H. seminalis* and were hence more conserved. The proteins of the transcription (K), replication, recombination, and repair (L), and the cell wall, membrane, and envelope biogenesis (M) formed the majority of the cloud genome. Significantly, the pangenome had a high proportion of cloud genes with hypothetical proteins. However, these unknown functional genes with limited distribution contributed to the genetic diversity of *H. seminalis*, and their biological roles require further research (Fig. S5; Data Set S1).

The pangenome and core genome of the analyzed genomes of *H*. *seminalis* are illustrated in Fig. 4B. As can be seen, the size of the core genome converged, and the pangenome size gradually expanded without reaching a plateau with the addition of new genomes. Furthermore, the fitting parameter B of the curve is greater than 0 and less than 1, which proved that the pangenome of *H. seminalis* is open. An open state of bacterial pangenome reflects the diversity within the gene pool of a given species.

### Potential virulence-associated gene prediction analyses.

**FIG 5:**
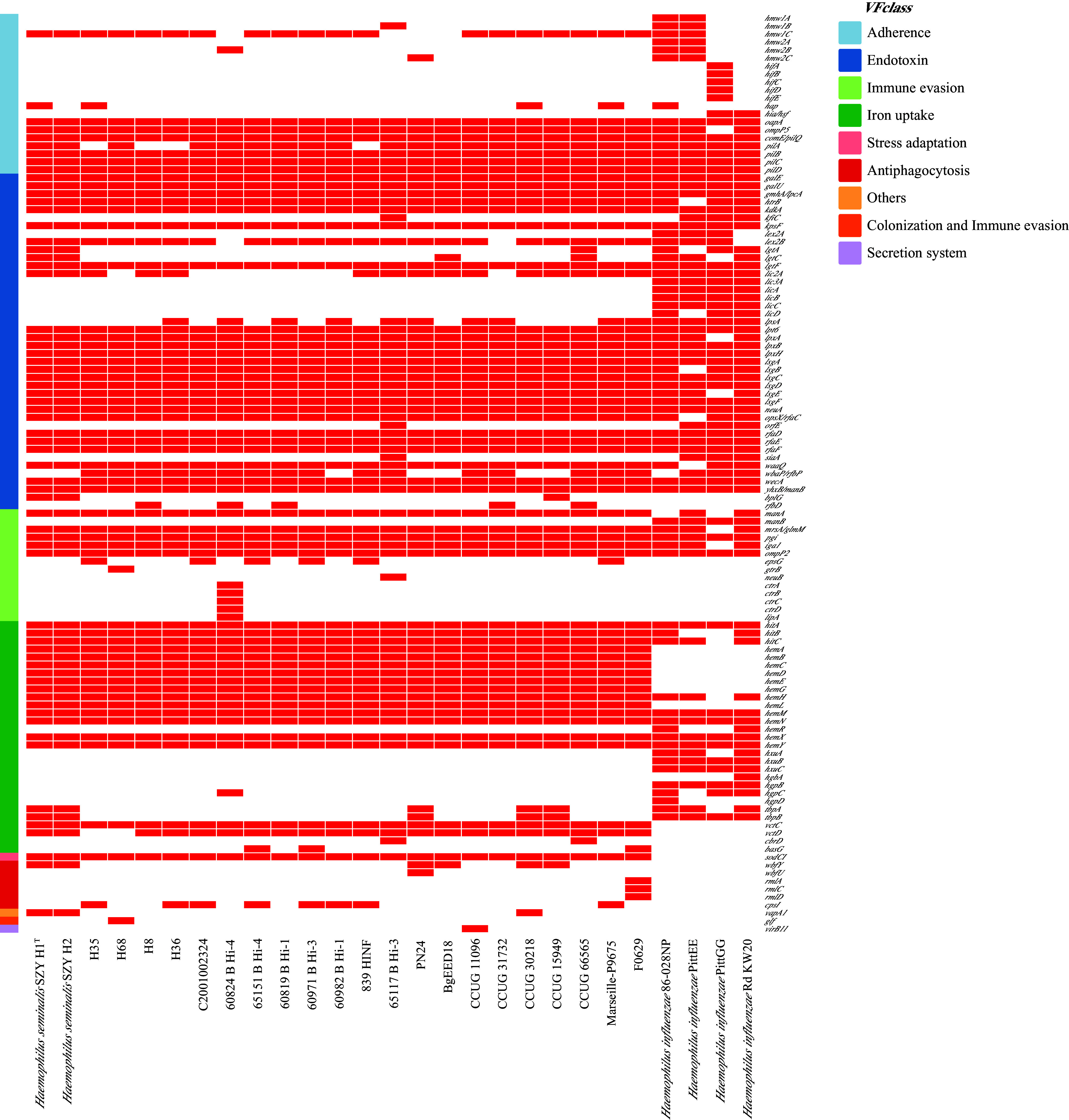
Virulence gene profiles of 23 *H. seminalis* and 4 H. influenzae isolates. The red square indicates the presence of the gene, and the white square indicates its absence. The legends on the right indicate the colors of the virulence factor class.

The virulence genes were predicted using a Virulence Factor Database (VFDB) analysis (17). The putative virulence genes of these 23 *H*. *seminalis* strains are presented in Fig. 5, and these genes were mainly associated with antiphagocytosis, adherence, immune evasion, endotoxin, and iron uptake (Data Set S1). Besides, the capsule-encoding genes *bexD*, *hcsA*, and *ccs1*, responsible for the synthesis of the polysaccharide capsule, were detected only in strain 60824 B Hi-4 (Table S6), while the genes *cpsI* and *epsG* could be found in some *H. seminalis* isolates of respiratory tract source. These genes were not detected in any of the six isolates from Guangzhou, nor were their capsular structures observed via transmission electron microscopy (Fig. S1).

Adherence to the host tissue is essential for bacterial colonization and subsequent infection (18). We observed that the hemagglutinating pili locus (*hifABCDE* genes), which is associated with biofilm formation (19), was not found in any of the 23 *H*. *seminalis* genomes. The *hap* gene, which encodes the adhesion and penetration Hap protein, was found in 4 of 23 (17.4%) *H*. *seminalis* genomes. The genes *hmw1c*, *oapA*, *ompP2*, *ompP5*, *comE/pilQ*, and *pilABCD*, which contribute to adhesion to host tissue, were also found in all 23 *H*. *seminalis* genomes. These genes encode HMW, OapA, OMP P2, OMP P5, and type IV pili, respectively. Notably, the multipurpose proteins of HMW, OMP P2, and OMP P5, with roles in adhesion and biofilm, also play an important role in internalization and immune evasion (20).

Immune exclusion is the most important defense mechanism involved in protection of mucosal membranes. The gene *iga1* encoding immunoglobulin A1 (IgA1) proteases, which is reported to be significantly correlated with the chronic obstructive pulmonary disease of NTHi isolates (21, 22), was found in all 23 *H*. *seminalis* genomes.

The lipooligosaccharide (LOS) of NTHi has been shown to be implicated in virulence and colonization (23). The 23 *H. seminalis* genomes shared a series of genes that are responsible for LOS synthesis: *galE*, *galU*, *gmhA/lpcA*, *htrB*, *kdkA*, *kpsF*, *lex2B*, *lgtF*, *lpt6*, *lpxA*, *lpxB*, *lpxH*, *lsgA*, *lsgB*, *lsgC*, *lsgD*, *lsgE*, *lsgF*, *neuA*, *opsX/rfaC*, *rfaD*, *rfaE*, *rfaF*, *waaQ*, *wbaP/rfbP*, and *wecA*. In comparison, the sialyltransferase genes *lic3A* and *lic3B* as well as the *siaA* genes were absent in these *H*. *seminalis* genomes. Interestingly, these three sialyltransferase genes were also absent in most of the H. haemolyticus genomes (24).

Despite limited research, exopolysaccharide (EPS) is also reported to be a component of the biofilm matrix for NTHi isolates (25). These genes (*manA*, *mrsA*/*glmM*, and *pgi* genes), which function in EPS synthesis, were found in most of the 23 *H*. *seminalis* genomes.

To promote persistence during infection, Haemophilus possesses the mechanisms to acquire iron and heme complex (26). The *hitABC* operon encoding a periplasmic-binding protein-dependent iron transport system was present in all *H. seminalis*, which is necessary for utilizing iron bound to transferrin or iron chelates (26). Notably, all *H. seminalis* strains have heme biosynthesis genes: *hemA*, *hemB*, *hemC*, *hemD*, *hemE*, *hemG*, and *hemN*. The derivatives of iron uptake genes, such as the *hgpABC* gene encoding a hemoglobin-binding protein, the *TbpAB* genes encoding a transferrin-binding protein, and the *HxuABC* genes encoding hemoglobin-binding complex, which are vital for acquiring iron and heme for growth, were not found in most of the 23 *H. seminalis* genomes.

### Antimicrobial susceptibility testing and antimicrobial resistance gene analyses.

The strains *H*. *seminalis* SZY H1^T^, SZY H2, SZY H8, SZY H35, SZY H36, and SZY H68 were susceptible to ampicillin/sulbactam, amoxicillin-clavulanic acid, piperacillin, piperacillin-tazobactam, cefotaxime, ceftriaxone, cefuroxime, cefepime, ceftazidime, aztreonam, meropenem, and imipenem, but they were resistant to tetracycline. The antimicrobial susceptibility results of the six *H*. *seminalis* isolates originating from Guangzhou are shown in Table S7.

The antimicrobial resistance genes were detected for the 23 *H*. *seminalis* genomes. The H. influenzae multidrug resistance (*hmrM*) gene was detected in all 23 (100%) isolates (Table S8). The cyclic AMP receptor protein (*CRP*) gene, which is related to antibiotic efflux, was detected in 20 (87.0%) isolates. However, the *tet*(*B*) gene, which confers resistance to tetracycline, was detected only in strain SZY H68 (4.3%). The *blaTEM-1* gene, which confers resistance to penicillins and first-generation cephalosphorins, was detected in 60824 B Hi-4 (4.3%).

## DISCUSSION

The genus Haemophilus is a heterogenous group and has been reported as a normal inhabitant of the upper respiratory tract of humans, the oral cavity, and mucous membranes (1–11). The new entry among the Haemophilus isolates is the description of two isolates with the name *H. seminalis* to indicate its isolation from the clinical semen samples of two patients who were suspected to be infected with Neisseria gonorrhoeae (9). This study further adds to the repertoires of Haemophilus isolates. This study provides a systemic characterization of four strains (SZY H8, SZY H35, SZY H36, and SZY H68) isolated from human sputum belonging to *H. seminalis*. Despite the low 16S rRNA gene sequence identity (<98.65%) to *H*. *seminalis* SZY H1^T^, they were classified to *H. seminalis* based on the comparative studies of phenotypic (Tables 2 and 3) and genotypic features, (e.g., ANI [>95%] values). To gain more insight into the *H. seminalis* strains, a comparative genomic analysis was conducted for 173 Haemophilus genomes retrieved from the NCBI GenBank database. These genomes include isolates previously identified as *H. seminalis*, H. haemolyticus, “*H*. *intermedius*”, “*H*. *quentini*”, and H. influenzae. 17 strains with >95% ANI values to SZY H1^T^ were finally identified, of which 14 were previously described as “*H. intermedius*” or hi*Hh* (11).

Historically, “*H. intermedius*” is informally named due to the intermediate position between H. influenzae and *H*. *parainfluenzae* (14). Although the previous study reclassified “*H*. *intermedius*” as hi*Hh* for their high genetic similarity to H. haemolyticus (11), we feel that the “*H*. *intermedius*” or hi*Hh* should be classified as *H*. *seminalis*. First, the name “*H. intermedius*”, was proposed at more or less the same time as *H*. *seminalis* and was not validated. Therefore, it was not recognized by ICSP, whereas the name *H. seminalis* is described as per the existing rules and regulations of ICNP. Second, the phylogenomic analyses demonstrated that the 14 “*H. intermedius*” or hi*Hh* isolates shared a highly homologous lineage with *H*. *seminalis* isolates, and this lineage is distinctive from the clades of the main H. haemolyticus group and that of H. influenzae isolates (Fig. 2). Third, the 14 “*H. intermedius*” or hi*Hh* isolates shared the same heme biosynthesis genes in their core genomes, similar to those of the *H. seminalis* isolates, but different from those of H. haemolyticus and H. influenzae. Therefore, we propose the reclassification of the isolates identified as “*H. intermedius*”, or hi*Hh* as belonging to *H. seminalis.* Accordingly, we propose an emendation of the description of the species of *H. seminalis*.

In recent years, a clear increase in urethral infections by Haemophilus spp. was observed, and the main route of transmission was reported to be unprotected oral sex and intercourse in men who have sex with men (7, 15, 27–30). Several Haemophilus species, for example, *H*. *parainfluenzae*, H. influenzae, “*H. quentini*”, *H*. *pittmaniae*, and *H*. *sputorum* have been reported to be the etiological agent of nongonococcal urethritis, of which the most common one was identified as Haemophilus parainfluenzae (7, 27, 29, 30). As the first isolation of *H*. *seminalis* was from the clinical semen samples, we hypothesized that these isolates are other pathogens responsible for causing reproductive tract infection. Unlike the earlier cases, the four isolates obtained in this study were from sputum samples of four immunocompromised patients with suspected tuberculosis, contact dermatitis, malignant tumor of the stomach, and nephrotic syndrome. Considering the relatedness of these four isolates to SZY H1^T^ from semen, as well as other isolates from bronchoalveolar lavage fluid, urine, eye, cerebrospinal fluid, feces, ascitic fluid, and pleural fluid, and its ability to grow like the other normal microbiota of upper respiratory tract, we define *H*. *seminalis* as a part of the human commensal flora that can cause invasive infections.

The comparative genomic analysis also reveals the genetic diversity and pathogenic potential of *H. seminalis* isolates. First, *H. seminalis* has an open pangenome. Microbes with an open pangenome and having large and diverse accessory genomes indicate that species can migrate to new niches and adapt to changing environmental conditions (31, 32). Second, *H. seminalis* was found to possess most of the virulence genes of H. influenzae, which implied a pathogenic potential similar to H. influenzae at a genomic level. For example, *hmw1c*, *oapA*, *ompP2*, *ompP5*, *comE/pilQ*, and *pilABCD* genes, which contributed to adhesion to host tissue for most NTHi isolates (33–37), were found in all the 23 *H*. *seminalis* genomes. The *iga1* gene that encoded specific IgAl proteases that cleave human IgA1, which is a significant protective gene of NTHi isolates (21, 22), was also found in all 23 *H*. *seminalis* genomes. The same goes for the series of genes that function in LOS and EPS synthesis; they are reported in most H. influenzae isolates (18, 21, 38) and were also found on almost all 23 *H*. *seminalis* genomes. However, the cellular capsule encoding locus of the *bexABCD*, *hcsA*, and *hcsB* genes, which is a major virulence factor of encapsulated typeable H. influenzae (39), was not present in the majority of the 23 *H*. *seminalis* genomes. As the cellular capsule was not observed, we infer that *H*. *seminalis* may represent a lineage with low virulence that is similar to *H. parainfluenzae*, H. haemolyticus, and nontypeable H. influenzae.

H. influenzae is known to acquire genetic material from other Haemophilus spp. (particularly H. haemolyticus), other unrelated species, and even host cells (12, 40, 41). Similarly, *H*. *seminalis* also has the competence to acquire genetic material from other species. The type IV pilus genes (*pilABCD*), which are necessary for competence, were found in all 23 *H*. *seminalis* genomes. The previous study demonstrated that the hemin biosynthesis loci acquired in the *H*. *seminalis* lineage were likely laterally transferred from an *H*. *parainfluenzae* ancestor (11).

Although the majority of Haemophilus isolates are noninfectious commensals, precise identification of *H*. *seminalis* among so many Haemophilus species is important when it grows dominantly in a valid sputum specimen. However, it is hard to distinguish *H*. *seminalis* from H. haemolyticus and H. influenzae using single phenotypic and genotypic methods. We attempted to assess *H*. *seminalis* intraspecies differences based on the 16S rRNA gene, 23S rRNA gene, and the housekeeping genes of *rpoB*, *recA*, *frdB*, *mdh*, *adk*, *atpG*, and *pgi*. The results show that these genes did not contain the sufficient differential capacity to effectively identify *H*. *seminalis* from H. haemolyticus and H. influenzae. Intraspecies classification was therefore undertaken using a whole-genome-based approach. Fortunately, the *ispD* gene was found to be a reliable phylogenetic marker for these three related species based on intraspecies comparisons. The rapid and reliable MALDI-TOF MS method for *H*. *seminalis* is also expected to be explored for species identification in clinical microbiology laboratories.

In this study, a pangenome analysis revealed that some housekeeping genes, such as *fabB*, *fabI*, *pal*, *ligA*, *folP*, and *rfaF*, were missing in individual strains of *H. seminalis*. However, the absence of these genes may be related to the limitations of whole-genome sequencing technology. Since short-read sequencing technology was used, it is likely that it influenced the number of contigs and the quality of the whole-genome assembly, leading to the missing of certain protein-coding genes in the genome analyses.

In conclusion, our study provides insights into the identification, epidemiology, genetic diversity, pathogenic potentials, and antimicrobial resistance of *H. seminalis*. Notably, the genome numbers in this study are not large enough to define a total accessory genome of *H. seminalis* and the addition of more high-quality genomes will probably increase the size of the accessory genes. Overall, a further epidemiological investigation is also expected to explore *H. seminalis* infection in the future.

## TAXONOMY

### Emended description of *Haemophilus seminalis* by Zheng et al., 2020.

The isolates previously described as “*H*. *intermedius*” or hi*Hh* should be included with the species description, as given by Zheng et al. (9), with the following amendments.

The cells were pleomorphic rods or coccobacilli. All tested strains grew between 28 to 40°C, although some isolates can grow at 42°C. The V-factor was dependent, but the X-factor was independent. Positive for catalase activity. Negative for motility. Varied for oxidase activity, hemolytic activity, nitrite reduction, and indole production. The G+C content ranged from 38.1% to 38.4%.

## MATERIALS AND METHODS

### Strains and genomes.

Strains SZY H1^T^ and SZY H2 were isolated from clinical human semen in our previous study (9). Four isolates, designated SZY H68, SZY H8, SZY H35, and SZY H36, were obtained from clinical sputum specimens of four hospitalized patients that were collected between September of 2018 and March of 2019 (Table 1). The genomic DNA of the four isolates was extracted using a bacterial genomic DNA extraction kit (AG, China), according to the manufacturer’s protocols, and then the whole-genome sequencing was performed by the Novogene Company (Beijing, People’s Republic of China).

We also examined, in total, 69 phylogenetically related publicly available genomes from GenBank (as of July of 2022), including 2 “*H. quentini”*, 14 “*H. intermedius*” or hi*Hh* (11, 14, 42, 43), 52 H. haemolyticus (44, 45), and 1 Haemophilus spp. in this study (Table 1; Table S2). In addition, the 98 complete genomes of H. influenzae, including 5 H. influenzae type a (Hia), 5 H. influenzae type b (Hib), 2 H. influenzae type c (Hic), 4 H. influenzae type e (Hie), 4 H. influenzae type f (Hif), and 78 nontypeable H. influenzae (NTHi), were used as references (Table S2).

### Phenotypic tests.

Gram-staining and acid-fast staining were carried out according to standard operating procedures. Morphological traits were observed by using transmission electron microscopy (JEM-1400, JEOL). H. influenzae ATCC 10211 was used as a capsulated control. Growth activity at 4, 8, 14, 28, 32, 35, 37, 40, and 42°C was observed by culturing the isolates on Haemophilus chocolate 2 agar at 35°C and 5% CO_2_. The requirements for X-factors and/or V-factors were assayed by using commercial discs supplemented with X-factors, V-factors, and X+V-factors (Liofilchem) on BHI agar. The satellite phenomenon and hemolytic activity were tested by observing the growth of the isolates around Staphylococcus aureus ATCC 25923 on Columbia blood agar. Traditional biochemical analyses, including the catalase, oxidase, arginine dihydrolase, urease, tryptophan decarboxylase, gelatinase, lysine decarboxylase activities, Simmons’ citrate, Voges-Proskauer, indole production, H_2_S production, hydrolysis of esculin, and reduction of nitrate, were assessed as described by Aslanzadeh et al. (46). Other physiological and biochemical characteristics were determined by using API NH and API ZYM kits, according to the manufacturer’s instructions (bioMérieux, France).

The biomass for the chemotaxonomic characterization of strains *H. seminalis* SZY H1^T^, SZY H2, SZY H68, SZY H8, SZY H35, SZY H36, H. haemolyticus CCUG 12834^T^, and H. influenzae ATCC 10211 was obtained from cultures grown on Haemophilus chocolate 2 agar for 2 days under 35°C and 5% CO_2_. Cellular fatty acid methyl esters were extracted and analyzed according to the procedure of the Microbial Identification System (MIDI, SherlockVersion6.3, TSBA6) (47). Polar lipids were extracted, separated, and analyzed via two-dimensional TLC, according to the method of Minnikin et al. (48).

Antimicrobial susceptibility testing was carried out on HTM agar (Detgerm, China) and a TDR NH-96 kit (Mindray, China). The susceptibility criterion was interpreted by referring to disc diffusion and MIC breakpoint using the MIC breakpoint of H. influenzae and *H. parainfluenzae* as the standard, as listed in the CLSI M100-S31 guidelines (Performance Standards for Antimicrobial Susceptibility Testing; Thirty-one Informational Supplement).

### Whole-genome sequencing, phylogenomic, and overall genome relatedness indices (OGRI) analyses.

The libraries of strains SZY H8, SZY H35, and SZY H36 were constructed using the Illumina PE150 platform (Novogene Company, Beijing, People’s Republic of China), and the library of strain SZY H68 was constructed using Nanopore and Illumina platform (Novogene Company, Beijing, People’s Republic of China). Second-generation high-throughput sequencing data were assembled by using SOAPdenovo version 2.04 (49), SPAdes (50), ABySS (51), CISA (52), and GapCloser version 1.12. Unicycler (53) was used to combine and assemble the PE150 data and Nanopore data. CheckM (v1.1.3) (54) was used to assess the quality of the assembled genomes. The genes that were extracted from the genomes were predicted and annotated by Prokka (v1.14) (55). COG functional annotation was performed with the eggNOG-mapper tool (56), based on the eggNOG database (http://eggnog5.embl.de/) (57). If a gene was assigned to two or more COG categories, each COG category was counted separately. Hicap (v1.0.3) (58) was utilized to identify and confirm the H. influenzae capsular serotypes.

The ANI values were generated and then visualized by using FastANI version 1.32 (59) and Chiplot (https://www.chiplot.online/). The dDDH was estimated by using the Genome-to-Genome Distance Calculator platform (https://ggdc.dsmz.de/distcalc2.php).

### rRNA and protein-encoding gene analysis.

The 16S rRNA genes of the four isolates were amplified and sequenced as previously described (9, 60). Meanwhile, the targeted rRNA and protein-encoding genes were extracted from the genome using RNAmmer (version 1.2) (61) and in-house JavaScript, respectively. The sequence comparison with those related strains was performed on the NCBI BLAST website (https://blast.ncbi.nlm.nih.gov/Blast.cgi). A maximum likelihood tree based on the nucleotide sequences was constructed for each of the mentioned genes. Multiple alignments were performed using MUSCLE (62). Poorly aligned regions were removed from the data sets via BMGE (63). A maximum likelihood (ML) phylogenomic tree was generated by the IQTree2 software (64), which automatically calculates the best-fit model. Bootstrap values were determined for 1,000 replications. Tree visualization was performed using iTOL (65).

### Pangenome analyses.

Through gff files produced by Prokka, pangenomes were deduced by using the pangenome pipeline Roary (66). Two sets of pangenomes were created. The first set contained all *H. seminalis* genomes. Pangenome analysis was performed on these strains genomes by using Roary software with the default blastP identity cutoff of 85%. The second contained 23 *H.seminalis*, 50 *H.haemolyticus*, 2 “*H.quentini*”, and 98 H. influenzae genomes. The core gene nucleotide sequences of these strains’ alignments were performed with Roary using PRANK (with MAFFT), and a blastP identity cutoff of 60%. FastTree v2.1.9 (67) was used to calculate maximum likelihood phylogenetic trees, based on these core gene alignments. The final tree was visualized using iTOL (65).

Related analysis data of the pangenome and core genome were obtained by using the method proposed by Knight et al. (68) and the models given by Tettelin et al. (69, 70). The curve fitting of the pangenome was performed using a power law regression based on Heaps’ Law (*y = Ax^B^ + C*), as previously described (69–71), where *y* is the pangenome size, *x* is the number of genomes, and *A*, *B*, and *C* are the fitting parameters. When 0 < *B* < 1, the size of the pangenome increases continuously with the sequential additions of new genomes, which indicates that the pangenome is open. In contrast, when *B* < 0 or *B* > 1, this shows that the pangenome is closed. Similar to the pangenome plot, the number of core genes after the addition of each new genome was plotted as a function of the number of genomes added in order. The exponential curve fit model (*y = Ae^Bx^ + C*) was used to fit the core genome (69–71), where *y* denotes the core genome size, *x* is the number of genomes, and *A*, *B*, and *C* are the fitting parameters. Both the pangenome and the core genome were visualized via PanGP (72).

### Virulence gene and antimicrobial resistance gene analysis.

The prediction of virulence genes was performed through searches querying each genome against the Virulence Factor Database (17) with the VFanalyzer web server. The default parameters were used. The results were visualized by using Chiplot. The detection of the capsule genes was performed by BLAST against a custom database of capsule genes, which was created based on a previous study (39). The resistance genes were identified using ABRicate v1.0.1 (https://github.com/tseemann/abricate) with the CARD database (73) and the Antibiotic Resistance Genes Database (ARDB) (http://ardb.cbcb.umd.edu/).

### Data availability.

The 16S rRNA of the SZY H8, SZY H35, SZY H36, and SZY H68 were deposited into GenBank with the accession numbers MZ191769 to MZ191772. The complete genome of SZY H68 and draft genomes of SZY H8, SZY H35, and SZY H36 have been deposited at DDBJ/ENA/GenBank under the accession numbers CP091469, JAHKJY000000000, JAHKJZ000000000, and JAHKKA000000000 (Table 1).
