# Supplementary material for: Comparative Genomic Analysis Reveals Genetic Diversity and Pathogenic Potential of Haemophilus seminalis and Emended Description of Haemophilus seminalis
Source: Microbiol Spectr. 2023 Jun 29;11(4):e04772-22. doi: 10.1128/spectrum.04772-22 (PMC10434262; doi:10.1128/spectrum.04772-22)
Supplement: Supplemental file 2 — Supplemental material. Download spectrum.04772-22-s0002.pdf, PDF file, 14.0 MB [file spectrum.04772-22-s0002.pdf]

Supplementary Material

Comparative Genomic Analysis Reveals Genetic Diversity and Pathogenic Potential of *Haemophilus seminalis*, and Emended Description of *Haemophilus seminalis*

Xiaowei Chen,[a] Hanyun Zhang,[b] Junhui Feng,[a] Lei Zhang,[c] Minling Zheng[d], Haimin Luo,[a,e] Huiyan Zhuo,[b] Ning Xu,[a,e] Xuan Zhang,[a,e] Cha Chen,[a,e] Pinghua Qu,[a,e#] Youqiang Li[b#]

[a]The Second Clinical College of Guangzhou University of Chinese Medicine, Guangzhou 510006, China
[b]Department of Laboratory Medicine, He Xian Memorial Hospital of Panyu District, Guangzhou 511400, China
[c]Guangzhou Kingmed Center for Clinical Laboratory, Guangzhou 510330, China
[d]Department of Clinical laboratory, Guangdong Women and Children Hospital, Guangzhou 511400, China
[e]Department of Clinical Laboratory, The Second Affiliated Hospital of Guangzhou University of Chinese Medicine, Guangdong Provincial Hospital of Chinese Medicine, Guangzhou 510006, China

Xiaowei Chen and Hanyun Zhang contributed equally to this work. Author order was determined by the corresponding authors after negotiation.
#Address correspondence to Pinghua Qu, ququtdr@163.com, or Youqiang Li, liyouqiang21@126.com.

Running Title: Genome-Based Diversity of *Haemophilus seminalis*

TABLE S1 Clinical data of the 4 isolates in this study.

Strain	Isolation date (dd/mm/yyyy)	Sex	Age	Specimen source	Culture result	Diagnosis	Comorbidity
SZY H68	26/09/2018	Female	21	Sputum	Normal microbiota of oropharynx	Pulmonary infection (suspected tuberculosis)	–
SZY H8	26/03/2019	Female	66	Sputum	Dominant growth of *H. haemolyticus*	Pulmonary infection	Contact dermatitis
SZY H35	01/09/2018	Male	75	Sputum	Normal microbiota of oropharynx	Chronic bronchitis with emphysema	Malignant tumor of stomach
SZY H36	24/09/2018	Male	73	Sputum	Normal microbiota of oropharynx	Chronic obstructive pulmonary disease	Nephrotic syndrome

TABEL S2 Related genomes used in this study.

No.	Species	Strain	Anatomical site	Country	genome size	GC%	Serotype	Accession number	Reference
1	*H. haemolyticus*	11P18	Nasopharynx	USA	1785806	38.40	–	LCTK00000000	(1)
2	*H. haemolyticus*	16-549009	Sputum	UK	1843335	38.50	–	RWKG00000000	–
3	*H. haemolyticus*	1P26	Nasopharynx	USA	1869699	38.50	–	LCTI00000000	(1)
4	*H. haemolyticus*	2019-19	Sputum	Japan	1895310	38.50	–	AP024093.1	(2)
5	*H. haemolyticus*	27P25	Nasopharynx	USA	1876155	38.20	–	LCTH00000000	(1)
6	*H. haemolyticus*	3P5	Nasopharynx	USA	1836392	38.40	–	LCTJ00000000	(1)
7	*H. haemolyticus*	ATCC 33390	Sputum	USA	1828364	38.60	–	JTLY00000000	–
8	*H. haemolyticus*	CCUG 24149	Vagina	Sweden	1941809	38.30	–	LZDL00000000	–
9	*H. haemolyticus*	CCUG 30047	Nasopharynx	Sweden	2100773	38.50	–	JACBKA00000000	–
10	*H. haemolyticus*	CCUG 39154	Eye	Sweden	1880185	38.30	–	LZOY00000000	–
11	*H. haemolyticus*	HI2028	Blood	USA	1848731	38.50	–	JTLX00000000	–
12	*H. haemolyticus*	HK386	–	USA	1822726	38.50	–	AJSV00000000	–
13	*H. haemolyticus*	M11818	–	–	1961876	38.30	–	QQHN00000000	(3)
14	*H. haemolyticus*	M19066	–	–	1845083	38.50	–	QQHH00000000	(3)
15	*H. haemolyticus*	M19071	–	–	1914164	38.30	–	QQJF00000000	(3)
16	*H. haemolyticus*	M19079	–	–	2010365	38.50	–	QQGY00000000	(3)
17	*H. haemolyticus*	M19080	–	–	1945186	38.30	–	QQIL00000000	(3)
18	*H. haemolyticus*	M19099	–	–	1874589	38.40	–	QQGD00000000	(3)
19	*H. haemolyticus*	M19107	–	–	1915087	38.70	–	AFQN00000000	(4)
20	*H. haemolyticus*	M19122	–	–	1979082	38.60	–	QQKM00000000	(3)
21	*H. haemolyticus*	M19135	–	–	1947390	38.40	–	QQIS00000000	(3)
22	*H. haemolyticus*	M19140	–	–	1912585	38.30	–	QQIR00000000	(3)
23	*H. haemolyticus*	M19155	–	–	1904880	38.50	–	QQIA00000000	(3)
24	*H. haemolyticus*	M19161	–	–	1915140	38.40	–	QQHM00000000	(3)
25	*H. haemolyticus*	M19164	–	–	1882562	38.40	–	QQFX00000000	(3)
26	*H. haemolyticus*	M19187	–	–	1795109	38.40	–	QQKA00000000	(3)
27	*H. haemolyticus*	M19197	–	–	1985988	38.40	–	QQMK00000000	(3)
28	*H. haemolyticus*	M19201	–	–	2079674	38.50	–	QQIY00000000	(3)
29	*H. haemolyticus*	M19345	–	–	1916320	38.50	–	CP031240.1	(3)

No.	Species	Strain	Anatomical site	Country	genome size	GC%	Serotype	Accession number	Reference
30	*H. haemolyticus*	M19346	–	–	1973061	38.50	–	CP031243.1	(3)
31	*H. haemolyticus*	M19501	–	–	1884911	38.50	–	AFQO00000000	(4)
32	*H. haemolyticus*	M19528	–	–	2041693	38.50	–	QQIN00000000	(3)
33	*H. haemolyticus*	M21127	–	–	1945624	38.60	–	AFQP00000000	(3)
34	*H. haemolyticus*	M21621	–	–	2089205	38.40	–	AFQQ00000000	(4)
35	*H. haemolyticus*	M25342	–	–	1865692	38.30	–	QQKH00000000	(3)
36	*H. haemolyticus*	M26156	–	–	1758988	38.50	–	QQGV00000000	(3)
37	*H. haemolyticus*	M26157	–	–	1872515	38.30	–	QQIX00000000	(3)
38	*H. haemolyticus*	M26160	–	–	1910682	38.40	–	QQIB00000000	(3)
39	*H. haemolyticus*	M26161	–	–	2065162	38.60	–	QQJW00000000	(3)
40	*H. haemolyticus*	M26164	–	–	1812369	38.50	–	QQHY00000000	(3)
41	*H. haemolyticus*	M26166	–	–	1992670	38.70	–	QQLP00000000	(3)
42	*H. haemolyticus*	M26167	–	–	1975598	38.30	–	QQKY00000000	(3)
43	*H. haemolyticus*	M26173	–	–	1945566	38.40	–	QQHG00000000	(3)
44	*H. haemolyticus*	M26174	–	–	1974928	38.80	–	QQKS00000000	(3)
45	*H. haemolyticus*	M26176	–	–	2026824	38.60	–	QQKD00000000	(3)
46	*H. haemolyticus*	M28486	–	–	1822569	38.60	–	CP031238.1	(3)
47	*H. haemolyticus*	M28908	–	–	1758067	38.60	–	QQHW00000000	(3)
48	*H. haemolyticus*	NCTC 10839	Nasopharynx	Denmark	1934644	38.50	–	LS483458.1	–
49	*H. haemolyticus*	M21639	–	–	2328161	38.60	–	AFQR00000000	(4)
50	*H. haemolyticus*	S32F2	Blood	China	2185767	38.50	–	JACBKB00000000	–
51	*'H. quentini'*	C860	Blood	Canada	2123195	38.50	–	MDJC00000000	–
52	*'H. quentini'*	MP1	Blood	UK	2151950	38.50	–	MCII00000000	(5)
53	*H. influenzae*	477	Middle ear	Finland	1846259	38.20	–	CP007470.1	(6)
54	*H. influenzae*	Hi375	Middle ear	Finland	1850897	38.20	–	CP009610.1	(7)
55	*H. influenzae*	C486	Middle ear	Finland	1846503	38.20	–	CP007471.1	(6)
56	*H. influenzae*	723	Middle ear	Finland	1887620	38.20	–	CP007472.1	(6)
57	*H. influenzae*	NCTC 8143	–	UK	1890645	38.20	–	LN831035.1	–
58	*H. influenzae*	NML-Hia-1	Blood	Canada	1829217	38.00	type a	CP017811.1	–
59	*H. influenzae*	FDAARGOS_199	Sputum	USA	1830681	38.10	–	CP020411.2	–
60	*H. influenzae*	48P106H1	Sputum	USA	1857175	38.00	–	CP020006.1	–

No.	Species	Strain	Anatomical site	Country	genome size	GC%	Serotype	Accession number	Reference
61	*H. influenzae*	48P153H1	Sputum	USA	1857048	38.00	–	CP020007.1	–
62	*H. influenzae*	5P28H1	Sputum	USA	1886450	38.20	–	CP020008.1	–
63	*H. influenzae*	5P54H1	Sputum	USA	1886411	38.20	–	CP020009.1	–
64	*H. influenzae*	67P38H1	Sputum	USA	1823096	38.00	–	CP020010.1	–
65	*H. influenzae*	67P56H1	Sputum	USA	1823172	38.00	–	CP020011.1	–
66	*H. influenzae*	6P24H2	Sputum	USA	1901558	38.10	–	CP020012.1	–
67	*H. influenzae*	6P32H1	Sputum	USA	1901533	38.10	–	CP020013.1	–
68	*H. influenzae*	11P6H	Sputum	USA	1813865	38.00	–	CP020014.1	–
69	*H. influenzae*	10P129H1	Sputum	USA	2047595	37.90	–	CP029620.1	(6)
70	*H. influenzae*	84P36H1	Sputum	USA	2025527	38.40	–	CP029621.1	(6)
71	*H. influenzae*	M21384	–	USA	1817261	38.10	type a	CP031250.1	(3)
72	*H. influenzae*	M21460	–	USA	1860196	38.10	–	CP031242.1	(3)
73	*H. influenzae*	M17648	–	USA	1816295	38.00	type c	CP031241.1	(3)
74	*H. influenzae*	M25588	–	USA	1848871	38.10	type a	CP031254.1	(3)
75	*H. influenzae*	M12125	–	USA	1804746	38.00	type c	CP031237.1	(3)
76	*H. influenzae*	M25267	–	USA	1914782	38.20	type e	CP031236.1	(3)
77	*H. influenzae*	M13034	–	USA	1887933	38.00	type e	CP031239.1	(3)
78	*H. influenzae*	M15895	–	USA	1919901	38.20	type e	CP031249.1	(3)
79	*H. influenzae*	P621-7028	Sputum	Spain	1838740	38.20	–	CP031689.1	(8)
80	*H. influenzae*	P650-8603	Sputum	Spain	1833710	38.10	–	CP031685.1	(8)
81	*H. influenzae*	P652-8881	Sputum	Spain	1811303	38.00	–	CP031684.1	(8)
82	*H. influenzae*	P669-6977	Sputum	Spain	2013003	38.40	–	CP031681.1	(8)
83	*H. influenzae*	P642-4396	Sputum	Spain	1897311	38.20	–	CP031686.1	(8)
84	*H. influenzae*	P679-2791	Sputum	Spain	1858634	38.10	–	CP031678.1	(8)
85	*H. influenzae*	P636-8296	Sputum	Spain	1840498	38.10	–	CP031688.1	(8)
86	*H. influenzae*	P676-2514	Sputum	Spain	1858630	38.10	–	CP031679.1	(8)
87	*H. influenzae*	P672-7661	Sputum	Spain	1833305	38.10	–	CP031680.1	(8)
88	*H. influenzae*	P665-7858	Sputum	Spain	1908143	38.10	–	CP031682.1	(8)
89	*H. influenzae*	P617-9224	Sputum	Spain	1848210	38.20	–	CP031690.1	(8)
90	*H. influenzae*	P595-8370	Sputum	Spain	1833864	38.10	–	CP031692.1	(8)
91	*H. influenzae*	P615-8618	Sputum	Spain	1840062	38.20	–	CP031691.1	(8)

No.	Species	Strain	Anatomical site	Country	genome size	GC%	Serotype	Accession number	Reference
92	*H. influenzae*	P641-4342	Sputum	Spain	1849483	38.10	–	CP031687.1	(8)
93	*H. influenzae*	P662-7189	Sputum	Spain	1904311	38.10	–	CP031683.1	(8)
94	*H. influenzae*	2018-Y40	Respiratory	Japan	1957393	38.20	–	AP022867.1	(9)
95	*H. influenzae*	TAMBA230	Blood	Japan	1809645	38.00	type a	AP022846.1	(10)
96	*H. influenzae*	M1C112_1	Cough swab	Australia	1894676	38.00	–	CP063127.1	(11)
97	*H. influenzae*	TA8730	–	Japan	1852308	38.10	type a	AP024414.1	–
98	*H. influenzae*	CHBN-II-1	Blood and spinal fluid	Japan	1979718	38.40	–	AP018764.1	–
99	*H. influenzae*	CHBN-II-2	Blood and spinal fluid	Japan	1979786	38.40	–	AP018765.1	–
100	*H. influenzae*	CHBN-II-3	Blood and spinal fluid	Japan	1853393	38.10	–	AP018766.1	–
101	*H. influenzae*	CHBN-II-4	–	Japan	1832515	38.10	–	AP018767.1	–
102	*H. influenzae*	CHBN-II-5	–	Japan	1805925	38.10	–	AP018768.1	–
103	*H. influenzae*	CHBN-II-6	–	Japan	1819364	38.10	–	AP018769.1	–
104	*H. influenzae*	CHBN-II-7	–	Japan	1886480	38.20	–	AP018770.1	–
105	*H. influenzae*	CHBN-II-8	–	Japan	1902536	38.10	–	AP018771.1	–
106	*H. influenzae*	CHBN-III-1	–	Japan	1816076	38.00	–	AP018772.1	–
107	*H. influenzae*	CHBN-III-2	–	Japan	1914722	38.20	–	AP018773.1	–
108	*H. influenzae*	CHBN-III-3	–	Japan	1810977	38.00	–	AP018774.1	–
109	*H. influenzae*	CHBN-III-4	–	Japan	1818266	38.00	–	AP018775.1	–
110	*H. influenzae*	CHBN-III-5	–	Japan	1822115	38.10	–	AP018776.1	–
111	*H. influenzae*	CHBN-III-6	–	Japan	1884503	38.00	–	AP018777.1	–
112	*H. influenzae*	CHBN-III-7	–	Japan	1911890	38.20	–	AP018778.1	–
113	*H. influenzae*	CHBN-III-8	–	Japan	1791343	38.00	–	AP018779.1	–
114	*H. influenzae*	CHBN-IV-1	–	Japan	1882642	38.10	–	AP018780.1	–
115	*H. influenzae*	CHBN-V-1	–	Japan	1953394	38.10	–	AP018781.1	–
116	*H. influenzae*	CHBN-V-2	–	Japan	1790924	38.00	–	AP018782.1	–
117	*H. influenzae*	CHBN-V-3	–	Japan	1936686	38.10	–	AP018783.1	–
118	*H. influenzae*	FDAARGOS_1479	Spinal fluid	USA	1902597	38.00	type b	CP082856.1	–
119	*H. influenzae*	FDAARGOS_1562	Sputum	South Korea	1842606	38.11	type f	CP085949.1	–
120	*H. influenzae*	FDAARGOS_1561	Blood and spinal fluid	USA	1834285	38.10	type f	CP085951.1	–
121	*H. influenzae*	FDAARGOS_1560	–	USA	1890662	38.20	–	CP085952.1	–
122	*H. influenzae*	NCTC 11931	–	–	2044007	38.00	–	LS483392.1	–

No.	Species	Strain	Anatomical site	Country	genome size	GC%	Serotype	Accession number	Reference
123	*H. influenzae*	NCTC 11426	–	UK	1834484	38.10	type f	LS483411.1	–
124	*H. influenzae*	NCTC 12975	–	–	1850791	38.10	–	LS483438.1	–
125	*H. influenzae*	NCTC 13377	–	–	1890469	38.00	type b	LS483480.1	–
126	*H. influenzae*	NCTC 12194	Nasopharynx	Denmark	1915356	38.20	–	LS483486.1	–
127	*H. influenzae*	NCTC 8455	–	–	1865137	38.10	type e	LS483496.1	–
128	*H. influenzae*	NCTC 11394	–	–	1860106	38.10	type b	LR134168.1	–
129	*H. influenzae*	NCTC 12699	–	–	1879945	38.00	–	LR134171.1	–
130	*H. influenzae*	NCTC 11873	–	–	1948880	38.10	–	LR134490.1	–
131	*H. influenzae*	NCTC 8468	Spinal fluid	–	1876886	38.10	type b	LR590465.1	–
132	*H. influenzae*	KR271	Blood	Sweden	1805497	38.10	–	OV040584.1	–
133	*H. influenzae*	3655	Middle ear	USA	1917048	38.20	–	OV040719.1	–
134	*H. influenzae*	10810	–	–	1981535	38.10	type b	FQ312006.1	–
135	*H. influenzae*	2019	–	–	1969659	38.30	–	CP008740.1	–
136	*H. influenzae*	86-028NP	Nasopharynx	USA	1914490	38.20	–	CP000057.2	(12)
137	*H. influenzae*	CGSHiCZ412602	Middle ear fluid	Czech	1811802	38.10	–	CP007805.1	–
138	*H. influenzae*	F3031	Eye	Brazil	1985832	38.20	–	FQ670178.1	(13)
139	*H. influenzae*	F3047	Eye	Brazil	2007018	38.20	–	FQ670204.1	(13)
140	*H. influenzae*	KR494	Blood	Sweden	1856176	38.10	type f	CP005967.1	(14)
141	*H. influenzae*	PittEE	Middle ear	USA	1813033	38.00	–	CP000671.1	(15)
142	*H. influenzae*	PittGG	Middle ear	USA	1887192	38.00	–	CP000672.1	(15)
143	*H. influenzae*	R2846	Middle ear secretions	USA	1819370	38.00	–	CP002276.1	(16)
144	*H. influenzae*	R2866	Blood	USA	1932306	38.10	–	CP002277.1	(16)
145	*H. influenzae*	Rd KW20	–	USA	1830138	38.20	–	L42023.1	(17)
146	*H. influenzae*	HE7/F1946	Skin	Brazil	1985844	38.20	–	CP043770.1	–
147	*H. influenzae*	HE40/F3043	Conjunctiva	Brazil	2000194	38.20	–	CP043811.1	–
148	*H. influenzae*	HE37/F3052	Conjunctiva	Brazil	1877864	38.10	–	CP043810.1	–
149	*H. influenzae*	HE24/F3037	Blood	Brazil	1987687	38.20	–	CP043772.1	–
150	*H. influenzae*	HE15/F3028	Cerebrospinal fluid	Brazil	1984979	38.20	–	CP043771.1	–

TABLE S3 The digital DNA-DNA hybridization (dDDH) values of 23 *H. seminalis* strains and related strains[a].

	1	2	3	4	5	6	7	8	9	10	11	12	13	14	15	16	17	18	19	20	21	22	23	24	25	26
1	100.00	99.80	98.40	88.20	86.40	74.70	74.70	73.40	73.40	71.70	71.90	69.70	70.50	69.90	67.30	67.00	64.20	64.50	65.10	65.10	66.40	65.30	63.30	52.20	50.50	40.70
2	99.80	100.00	98.50	88.20	86.40	74.70	74.70	73.40	73.50	71.70	72.00	69.70	70.50	69.90	67.30	67.00	64.20	64.50	65.10	65.10	66.40	65.30	63.30	52.20	50.50	40.70
3	98.40	98.50	100.00	87.70	86.20	74.80	74.60	73.40	73.70	72.10	71.90	69.50	70.30	70.00	67.20	67.10	64.40	64.50	65.00	65.00	66.40	65.50	63.50	52.40	50.60	40.70
4	88.20	88.20	87.70	100.00	83.70	75.40	75.40	76.00	74.10	71.50	71.40	69.50	70.10	69.50	67.60	67.10	63.60	64.60	65.40	65.40	66.40	65.20	63.70	52.80	50.70	40.70
5	86.40	86.40	86.20	83.70	100.00	75.70	75.00	73.20	74.80	70.90	71.90	69.50	70.40	70.20	67.30	67.70	63.90	64.90	65.60	65.70	66.10	65.80	64.10	52.50	50.90	41.10
6	74.70	74.70	74.80	75.40	75.70	100.00	98.60	83.60	82.10	71.30	70.60	71.10	70.40	68.90	67.40	67.40	63.60	64.80	65.50	65.50	67.10	64.70	63.30	52.60	50.40	41.00
7	74.70	74.70	74.60	75.40	75.00	98.60	100.00	84.00	81.80	71.10	70.90	71.60	70.40	69.00	67.40	67.70	63.90	65.00	65.40	65.40	67.20	64.70	63.40	52.70	50.50	40.90
8	73.40	73.40	73.40	76.00	73.20	83.60	84.00	100.00	80.20	70.50	71.40	70.40	70.20	69.50	66.60	67.30	63.60	64.10	65.30	65.30	68.50	65.20	63.00	52.80	50.40	40.80
9	73.40	73.50	73.70	74.10	74.80	82.10	81.80	80.20	100.00	71.80	71.10	70.70	70.20	69.70	67.10	67.70	63.80	64.90	65.20	65.20	66.90	65.00	63.10	52.60	50.50	40.80
10	71.70	71.70	72.10	71.50	70.90	71.30	71.10	70.50	71.80	100.00	80.80	72.00	70.30	69.60	66.70	68.10	63.60	64.70	65.40	65.40	67.00	65.50	63.00	52.60	50.50	41.00
11	71.90	72.00	71.90	71.40	71.90	70.60	70.90	71.40	71.10	80.80	100.00	70.60	69.80	69.70	67.20	67.80	63.80	64.60	65.10	65.10	66.80	65.70	63.50	52.90	51.00	41.20
12	69.70	69.70	69.50	69.50	69.50	71.10	71.60	70.40	70.70	72.00	70.60	100.00	70.40	70.30	67.40	67.50	64.70	65.60	64.90	64.90	66.10	65.30	64.10	52.40	50.80	41.40
13	70.50	70.50	70.30	70.10	70.40	70.40	70.40	70.20	70.20	70.30	69.80	70.40	100.00	72.20	67.90	67.10	65.20	65.70	65.60	65.60	66.20	65.40	64.40	51.90	50.90	41.00
14	69.90	69.90	70.00	69.50	70.20	68.90	69.00	69.50	69.70	69.60	69.70	70.30	72.20	100.00	67.30	68.30	63.80	65.90	64.60	64.60	65.50	65.10	63.70	52.10	51.00	40.90
15	67.30	67.30	67.20	67.60	67.30	67.40	67.40	66.60	67.10	66.70	67.20	67.40	67.90	67.30	100.00	66.20	65.80	66.10	65.40	65.40	65.60	65.50	64.70	52.60	50.70	41.30
16	67.00	67.00	67.10	67.10	67.70	67.40	67.70	67.30	67.70	68.10	67.80	67.50	67.10	68.30	66.20	100.00	64.50	65.70	65.40	65.40	65.90	65.50	64.80	52.90	50.80	40.80
17	64.20	64.20	64.40	63.60	63.90	63.60	63.90	63.60	63.80	63.60	63.80	64.70	65.20	63.80	65.80	64.50	100.00	66.40	64.60	64.60	64.10	65.10	65.70	52.80	51.30	41.20
18	64.50	64.50	64.50	64.60	64.90	64.80	65.00	64.10	64.90	64.70	64.60	65.60	65.70	65.90	66.10	65.70	66.40	100.00	65.20	65.20	64.90	65.50	65.90	52.90	51.90	41.20
19	65.10	65.10	65.00	65.40	65.60	65.50	65.40	65.30	65.20	65.40	65.10	64.90	65.60	64.60	65.40	65.40	64.60	65.20	100.00	100.00	77.40	71.80	65.10	53.20	51.30	41.20
20	65.10	65.10	65.00	65.40	65.70	65.50	65.40	65.30	65.20	65.40	65.10	64.90	65.60	64.60	65.40	65.40	64.60	65.20	100.00	100.00	77.40	71.80	65.10	53.20	51.30	41.20
21	66.40	66.40	66.40	66.40	66.10	67.10	67.20	68.50	66.90	67.00	66.80	66.10	66.20	65.50	65.60	65.90	64.10	64.90	77.40	77.40	100.00	72.90	64.90	52.80	51.20	40.80
22	65.30	65.30	65.50	65.20	65.80	64.70	64.70	65.20	65.00	65.50	65.70	65.30	65.40	65.10	65.50	65.50	65.10	65.50	71.80	71.80	72.90	100.00	66.00	53.40	51.40	41.00
23	63.30	63.30	63.50	63.70	64.10	63.30	63.40	63.00	63.10	63.00	63.50	64.10	64.40	63.70	64.70	64.80	65.70	65.90	65.10	65.10	64.90	66.00	100.00	53.80	51.50	41.40
24	52.20	52.20	52.40	52.80	52.50	52.60	52.70	52.80	52.60	52.60	52.90	52.40	51.90	52.10	52.60	52.90	52.80	52.90	53.20	53.20	52.80	53.40	53.80	100.00	59.30	43.80
25	50.50	50.50	50.60	50.70	50.90	50.40	50.50	50.40	50.50	50.50	51.00	50.80	50.90	51.00	50.70	50.80	51.30	51.90	51.30	51.30	51.20	51.40	51.50	59.30	100.00	43.90
26	40.70	40.70	40.70	40.70	41.10	41.00	40.90	40.80	40.80	41.00	41.20	41.40	41.00	40.90	41.30	40.80	41.20	41.20	41.20	41.20	40.80	41.00	41.40	43.80	43.90	100.00

[a]Strains are indicated as 1, 65151 B Hi-4; 2, 60971 B Hi-3; 3, C2001002324; 4, SZY H35; 5, SZY H68; 6, Marseille-P9675; 7, 839 HINF; 8, CCUG 11096; 9, SZY H8; 10, CCUG 66565; 11, CCUG 31732; 12, F0629 CP027235; 13, SZY H36; 14, 60982 B Hi-1; 15, 60819 B Hi-1; 16, BgEED18; 17, 65117 B Hi-3; 18, PN24; 19, SZY H2; 20, SZY H1^{T}; 21, CCUG 30218; 22, CCUG 15949; 23, 60824 B Hi-4; 24, *H. haemolyticus* ATCC 33390^{T}; 25, '*H. quentini*' MP1; 26, *H. influenzae* NCTC 8143^{T}.

TABLE S4 Nucleotide identities (%) of 16S rRNA gene sequences between *H. seminalis* strains and related strains[a].

	1	2	3	4	5	6	7	8	9	10	11	12	13	14	15	16	17	18	19	20	21	22	23
1	100.00	99.80	99.78	98.91	98.47	98.45	98.02	98.02	97.75	97.61	97.61	97.47	97.47	97.47	97.47	97.34	97.34	97.30	98.13	97.82	99.04	97.95	95.97
2	99.80	100.00	99.93	99.11	98.65	98.64	98.22	98.23	97.95	97.81	97.81	97.68	97.68	97.68	97.68	97.54	97.54	97.47	98.34	98.02	99.25	98.15	96.18
3	99.78	99.93	100.00	99.06	98.67	98.39	98.04	98.12	97.80	97.61	97.61	97.54	97.54	97.54	97.51	97.37	97.36	97.50	98.14	97.83	99.13	98.09	96.02
4	98.91	99.11	99.06	100.00	99.31	99.24	98.82	98.30	98.63	98.50	98.63	98.43	98.43	98.43	98.36	98.22	98.22	98.29	98.77	97.98	99.87	97.95	96.35
5	98.47	98.65	98.67	99.31	100.00	100.00	99.48	97.58	98.20	98.61	98.44	98.79	98.79	98.79	98.74	98.56	98.65	98.70	98.07	97.93	99.31	98.74	96.98
6	98.45	98.64	98.39	99.24	100.00	100.00	99.71	97.54	98.45	98.00	98.00	98.48	98.48	98.48	98.44	98.45	98.45	98.00	98.10	97.25	99.33	97.47	95.65
7	98.02	98.22	98.04	98.82	99.48	99.71	100.00	97.46	98.02	98.23	98.17	98.43	98.43	98.43	98.36	98.22	98.36	98.21	97.74	97.59	98.89	97.74	96.80
8	98.02	98.23	98.12	98.30	97.58	97.54	97.46	100.00	99.18	98.76	98.69	98.82	98.82	98.82	98.77	98.64	98.64	98.62	99.11	99.28	98.30	97.07	97.13
9	97.75	97.96	97.81	98.63	98.20	98.45	98.02	99.18	100.00	99.18	99.11	99.59	99.59	99.59	99.59	99.45	99.45	99.41	99.58	98.57	98.63	96.99	96.73
10	97.61	97.82	97.61	98.50	98.62	98.00	98.24	98.76	99.18	100.00	99.80	99.48	99.48	99.48	99.45	99.32	99.32	99.76	99.45	99.22	98.56	96.86	96.94
11	97.61	97.82	97.61	98.63	98.44	98.00	98.17	98.69	99.11	99.80	100.00	99.35	99.35	99.35	99.32	99.18	99.18	99.59	99.38	99.09	98.63	97.06	96.81
12	97.48	97.68	97.54	98.43	98.79	98.48	98.43	98.82	99.59	99.48	99.35	100.00	100.00	100.00	100.00	99.86	99.86	99.92	99.18	99.02	98.37	97.20	97.26
13	97.48	97.68	97.54	98.43	98.79	98.48	98.43	98.82	99.59	99.48	99.35	100.00	100.00	100.00	100.00	99.86	99.86	99.92	99.18	99.02	98.37	97.20	97.26
14	97.48	97.68	97.54	98.43	98.79	98.48	98.43	98.82	99.59	99.48	99.35	100.00	100.00	100.00	100.00	99.86	99.86	99.92	99.18	99.02	98.37	97.20	97.26
15	97.47	97.68	97.52	98.36	98.74	98.45	98.36	98.77	99.59	99.45	99.32	100.00	100.00	100.00	100.00	99.86	99.86	99.92	99.17	98.98	98.29	97.19	97.14
16	97.34	97.55	97.37	98.22	98.56	98.45	98.23	98.64	99.45	99.32	99.18	99.86	99.86	99.86	99.86	100.00	99.73	99.75	99.03	98.84	98.16	97.07	97.01
17	97.34	97.55	97.37	98.22	98.65	98.45	98.36	98.64	99.45	99.32	99.18	99.86	99.86	99.86	99.86	99.73	100.00	100.00	99.03	98.98	98.16	97.20	97.01
18	97.30	97.47	97.50	98.29	98.70	98.01	98.21	98.62	99.41	99.76	99.59	99.92	99.92	99.92	99.92	99.75	100.00	100.00	99.31	99.27	98.29	97.72	97.33
19	98.13	98.34	98.14	98.77	98.07	98.10	97.74	99.11	99.58	99.45	99.38	99.18	99.18	99.18	99.17	99.03	99.03	99.31	100.00	98.91	98.91	96.81	96.46
20	97.82	98.02	97.83	97.98	97.93	97.25	97.59	99.28	98.57	99.22	99.09	99.02	99.02	99.02	98.98	98.84	98.98	99.27	98.91	100.00	98.04	97.07	97.39
21	99.04	99.25	99.13	99.87	99.31	99.33	98.89	98.30	98.63	98.56	98.63	98.36	98.36	98.36	98.29	98.15	98.15	98.29	98.90	98.04	100.00	97.81	96.35
22	97.95	98.15	98.09	97.95	98.74	97.47	97.74	97.07	96.99	96.85	97.06	97.20	97.20	97.20	97.19	97.06	97.20	97.72	96.81	97.06	97.81	100.00	95.91
23	95.97	96.18	96.02	96.35	96.98	95.65	96.80	97.13	96.73	96.94	96.81	97.26	97.26	97.26	97.14	97.01	97.01	97.33	96.46	97.39	96.35	95.91	100.00

[a]Strains are indicated as 1, SZY H1^T; 2, SZY H2; 3, CCUG 15949; 4, PN24; 5, CCUG 66565; 6, CCUG 31732; 7, F0629; 8, BgEED18; 9, SZY H36; 10, 65117 B Hi-3; 11, 60824 B Hi-4; 12, 60971 B Hi-3; 13, 65151 B Hi-4; 14, C2001002324; 15, SZY H35; 16, SZY H68; 17, SZY H8; 18, 839 HINF; 19, 60819 B Hi-1; 20, Marseille-P9675; 21, *H. haemolyticus* ATCC 33390^T; 22, *'H. quentini'*MCCM 02026; 23, *H. influenzae* NCTC 8143^T

TABLE S5 Pairwise *ispD* gene sequence identities values (%) of the 23 *H. seminalis* strains and related strains[a].

	1	2	3	4	5	6	7	8	9	10	11	12	13	14	15	16	17	18	19	20	21	22	23	24	25	26
1	100.00	100.00	98.38	98.97	98.23	97.79	97.79	97.35	97.49	97.64	98.08	97.94	98.38	98.08	98.08	98.08	98.08	98.08	97.64	98.08	98.08	97.79	97.79	77.75	77.42	76.95
2	100.00	100.00	98.38	98.97	98.23	97.79	97.79	97.35	97.49	97.64	98.08	97.94	98.38	98.08	98.08	98.08	98.08	98.08	97.64	98.08	98.08	97.79	97.79	77.75	77.42	76.95
3	98.38	98.38	100.00	99.12	99.85	97.64	97.64	97.79	97.64	97.79	97.94	97.79	98.82	97.94	97.94	97.94	97.94	97.94	97.49	97.94	98.23	97.64	97.64	77.60	76.91	76.51
4	98.97	98.97	99.12	100.00	98.97	97.64	97.64	97.79	97.64	97.79	97.94	97.79	99.12	97.94	97.94	97.94	97.94	97.94	97.49	97.94	98.23	97.64	97.64	77.75	77.42	76.65
5	98.23	98.23	99.85	98.97	100.00	97.64	97.49	97.79	97.64	97.64	97.79	97.64	98.67	97.79	97.79	97.79	97.79	97.79	97.49	97.79	98.08	97.64	97.64	77.60	76.91	76.51
6	97.79	97.79	97.64	97.64	97.64	100.00	98.53	98.97	98.53	98.38	99.41	98.97	97.79	99.41	99.41	99.41	99.41	99.41	99.85	99.41	98.82	100.00	100.00	77.86	77.60	76.39
7	97.79	97.79	97.64	97.64	97.49	98.53	100.00	98.08	98.82	98.67	98.82	98.97	97.64	98.82	98.82	98.82	98.82	98.82	98.38	98.82	99.12	98.53	98.53	77.78	77.60	76.90
8	97.35	97.35	97.79	97.79	97.79	98.97	98.08	100.00	98.38	98.23	98.97	98.82	97.94	98.97	98.97	98.97	98.97	98.97	98.82	98.97	98.67	98.97	98.97	77.78	77.45	76.54
9	97.49	97.49	97.64	97.64	97.64	98.53	98.82	98.38	100.00	98.67	98.53	98.67	97.64	98.53	98.53	98.53	98.53	98.53	98.38	98.53	99.12	98.53	98.53	77.99	77.60	76.69
10	97.64	97.64	97.79	97.79	97.64	98.38	98.67	98.23	98.67	100.00	98.97	98.82	97.79	98.97	98.97	98.97	98.97	98.97	98.23	98.97	99.26	98.38	98.38	77.92	77.60	76.83
11	98.08	98.08	97.94	97.94	97.79	99.41	98.82	98.97	98.53	98.97	100.00	99.26	98.08	100.00	100.00	100.00	100.00	100.00	99.26	100.00	99.12	99.41	99.41	77.92	77.60	76.69
12	97.94	97.94	97.79	97.79	97.64	98.97	98.97	98.82	98.67	98.82	99.26	100.00	97.94	99.26	99.26	99.26	99.26	99.26	98.82	99.26	99.56	98.97	98.97	77.63	77.31	76.54
13	98.38	98.38	98.82	99.12	98.67	97.79	97.64	97.94	97.64	97.79	98.08	97.94	100.00	98.08	98.08	98.08	98.08	98.08	97.64	98.08	98.23	97.79	97.79	77.75	77.42	76.65
14	98.08	98.08	97.94	97.94	97.79	99.41	98.82	98.97	98.53	98.97	100.00	99.26	98.08	100.00	100.00	100.00	100.00	100.00	99.26	100.00	99.12	99.41	99.41	77.92	77.60	76.69
15	98.08	98.08	97.94	97.94	97.79	99.41	98.82	98.97	98.53	98.97	100.00	99.26	98.08	100.00	100.00	100.00	100.00	100.00	99.26	100.00	99.12	99.41	99.41	77.92	77.60	76.69
16	98.08	98.08	97.94	97.94	97.79	99.41	98.82	98.97	98.53	98.97	100.00	99.26	98.08	100.00	100.00	100.00	100.00	100.00	99.26	100.00	99.12	99.41	99.41	77.92	77.60	76.69
17	98.08	98.08	97.94	97.94	97.79	99.41	98.82	98.97	98.53	98.97	100.00	99.26	98.08	100.00	100.00	100.00	100.00	100.00	99.26	100.00	99.12	99.41	99.41	77.92	77.60	76.69
18	98.08	98.08	97.94	97.94	97.79	99.41	98.82	98.97	98.53	98.97	100.00	99.26	98.08	100.00	100.00	100.00	100.00	100.00	99.26	100.00	99.12	99.41	99.41	77.92	77.60	76.69
19	97.64	97.64	97.49	97.49	97.49	99.85	98.38	98.82	98.38	98.23	99.26	98.82	97.64	99.26	99.26	99.26	99.26	99.26	100.00	99.26	98.67	99.85	99.85	78.01	77.75	76.54
20	98.08	98.08	97.94	97.94	97.79	99.41	98.82	98.97	98.53	98.97	100.00	99.26	98.08	100.00	100.00	100.00	100.00	100.00	99.26	100.00	99.12	99.41	99.41	77.92	77.60	76.69
21	98.08	98.08	98.23	98.23	98.08	98.82	99.12	98.67	99.12	99.26	99.12	99.56	98.23	99.12	99.12	99.12	99.12	99.12	98.67	99.12	100.00	98.82	98.82	77.63	77.31	76.54
22	97.79	97.79	97.64	97.64	97.64	100.00	98.53	98.97	98.53	98.38	99.41	98.97	97.79	99.41	99.41	99.41	99.41	99.41	99.85	99.41	98.82	100.00	100.00	77.86	77.60	76.39
23	97.79	97.79	97.64	97.64	97.64	100.00	98.53	98.97	98.53	98.38	99.41	98.97	97.79	99.41	99.41	99.41	99.41	99.41	99.85	99.41	98.82	100.00	100.00	77.86	77.60	76.39
24	77.75	77.75	77.60	77.75	77.60	77.86	77.78	77.78	77.99	77.92	77.92	77.63	77.75	77.92	77.92	77.92	77.92	77.92	78.01	77.92	77.63	77.86	77.86	100.00	96.46	90.27
25	77.35	77.35	76.91	77.35	76.98	77.53	77.53	77.39	77.53	77.53	77.53	77.24	77.35	77.53	77.53	77.53	77.53	77.53	77.68	77.53	77.24	77.53	77.53	96.46	100.00	90.27
26	76.95	76.95	76.51	76.65	76.57	76.39	76.90	76.54	76.69	76.83	76.69	76.54	76.65	76.69	76.69	76.69	76.69	76.69	76.54	76.69	76.54	76.39	76.39	90.27	90.27	100.00

[a]Strains are indicated as 1, SZY H1^{T}; 2, SZY H2; 3, CCUG 15949; 4, PN24; 5, CCUG 66565; 6, CCUG 31732; 7, 60982 B Hi-1; 8, F0629; 9, BgEED18; 10, SZY H36; 11, Marseille-P9675; 12, 65117 B Hi-3; 13, 60824 B Hi-4; 14, 60971 B Hi-3; 15, 65151 B Hi-4; 16, C2001002324; 17, SZY H35; 18, SZY H68; 19, SZY H8; 20, 839 HINF; 21, 60819 B Hi-1; 22, CCUG 30218; 23, CCUG 11096; 24, *H. haemolyticus* ATCC 33390^{T}; 25, '*H. quentini*' MP1; 26, *H. influenzae* NCTC 8143^{T}.

TABLE S6 Detection of capsule genes in the 23 *H. seminalis* strains[a].

Gene	1	2	3	4	5	6	7	8	9	10	11	12	13	14	15	16	17	18	19	20	21	22	23	Total
bcs2	–	–	–	–	–	–	–	–	–	–	–	–	–	–	–	–	–	–	–	–	–	–	–	0
bcs3	–	–	–	–	–	–	–	–	–	–	–	–	–	–	–	–	–	–	–	–	–	–	–	0
bcs4	–	–	–	–	–	–	–	–	–	–	–	–	–	–	–	–	–	–	–	–	–	–	–	0
bexA	–	–	–	–	–	–	–	–	–	–	–	–	–	–	–	–	–	–	–	–	–	–	–	0
bexB	–	–	–	–	–	–	–	–	–	–	–	–	–	–	–	–	–	–	–	–	–	–	–	0
bexC	–	–	–	–	–	–	–	–	–	–	–	–	–	–	–	–	–	–	–	–	–	–	–	0
bexD	–	–	–	–	–	–	–	+	–	–	–	–	–	–	–	–	–	–	–	–	–	–	–	1
ccs1	–	–	–	–	–	–	–	+	–	–	–	–	–	–	–	–	–	–	–	–	–	–	–	1
ccs2	–	–	–	–	–	–	–	–	–	–	–	–	–	–	–	–	–	–	–	–	–	–	–	0
ccs3	–	–	–	–	–	–	–	–	–	–	–	–	–	–	–	–	–	–	–	–	–	–	–	0
ccs4	–	–	–	–	–	–	–	–	–	–	–	–	–	–	–	–	–	–	–	–	–	–	–	0
dcs1	–	–	–	–	–	–	–	–	–	–	–	–	–	–	–	–	–	–	–	–	–	–	–	0
dcs2	–	–	–	–	–	–	–	–	–	–	–	–	–	–	–	–	–	–	–	–	–	–	–	0
dcs3	–	–	–	–	–	–	–	–	–	–	–	–	–	–	–	–	–	–	–	–	–	–	–	0
dcs4	–	–	–	–	–	–	–	–	–	–	–	–	–	–	–	–	–	–	–	–	–	–	–	0
dcs5	–	–	–	–	–	–	–	–	–	–	–	–	–	–	–	–	–	–	–	–	–	–	–	0
ecs1	–	–	–	–	–	–	–	–	–	–	–	–	–	–	–	–	–	–	–	–	–	–	–	0
ecs2	–	–	–	–	–	–	–	–	–	–	–	–	–	–	–	–	–	–	–	–	–	–	–	0
ecs3	–	–	–	–	–	–	–	–	–	–	–	–	–	–	–	–	–	–	–	–	–	–	–	0
ecs4	–	–	–	–	–	–	–	–	–	–	–	–	–	–	–	–	–	–	–	–	–	–	–	0
ecs5	–	–	–	–	–	–	–	–	–	–	–	–	–	–	–	–	–	–	–	–	–	–	–	0
ecs6	–	–	–	–	–	–	–	–	–	–	–	–	–	–	–	–	–	–	–	–	–	–	–	0
ecs7	–	–	–	–	–	–	–	–	–	–	–	–	–	–	–	–	–	–	–	–	–	–	–	0
ecs8	–	–	–	–	–	–	–	–	–	–	–	–	–	–	–	–	–	–	–	–	–	–	–	0
fcs1	–	–	–	–	–	–	–	–	–	–	–	–	–	–	–	–	–	–	–	–	–	–	–	0
fcs2	–	–	–	–	–	–	–	–	–	–	–	–	–	–	–	–	–	–	–	–	–	–	–	0
fcs3	–	–	–	–	–	–	–	–	–	–	–	–	–	–	–	–	–	–	–	–	–	–	–	0
hcsA	–	–	–	–	–	–	–	+	–	–	–	–	–	–	–	–	–	–	–	–	–	–	–	1
hcsB	–	–	–	–	–	–	–	–	–	–	–	–	–	–	–	–	–	–	–	–	–	–	–	0
acs1	–	–	–	–	–	–	–	–	–	–	–	–	–	–	–	–	–	–	–	–	–	–	–	0
acs2	–	–	–	–	–	–	–	–	–	–	–	–	–	–	–	–	–	–	–	–	–	–	–	0
acs3	–	–	–	–	–	–	–	–	–	–	–	–	–	–	–	–	–	–	–	–	–	–	–	0
acs4	–	–	–	–	–	–	–	–	–	–	–	–	–	–	–	–	–	–	–	–	–	–	–	0
bcs1	–	–	–	–	–	–	–	–	–	–	–	–	–	–	–	–	–	–	–	–	–	–	–	0
Total	0	0	0	0	0	0	0	3	0	0	0	0	0	0	0	0	0	0	0	0	0	0	0	3

[a]Strains are indicated as 1, SZY H1^{T}; 2, SZY H2; 3, H35; 4, H68; 5, H8; 6, H36; 7, C2001002324; 8, 60824 B Hi-4 ; 9, 65151 B Hi-4; 10, 60819 B Hi-1; 11, 60971 B Hi-3 ; 12, 60982 B Hi-1; 13, 839 HINF; 14, 65117 B Hi-3; 15, PN24; 16, BgEED18; 17, CCUG 11096; 18, CCUG 31732; 19, CCUG 30218; 20, CCUG 15949; 21, CCUG 66565; 22, Marseille-P9675; 23, F0629. –, not detected; +, detected.

TABLE S7 Antibiotic susceptibility of strains SZY H68, SZY H8, SZY H35, SZY H36, and their related strains[a].

Antibiotic	1	2	3	4	5	6	7
Sulbactam/ampicillin	S	S	S	S	S	S	R
Amoxycillin/clavulanic acid	S	S	S	S	S	S	S
Piperacillin	S	S	S	S	S	S	S
Piperacillin/tazobactam	S	S	S	S	S	S	S
Cefotaxime	S	S	S	S	S	S	S
Ceftriaxone	S	S	S	S	S	S	S
Cefuroxime sodium	S	S	S	S	S	S	I
Cefepime	S	S	S	S	S	S	S
Ceftazidime	S	S	S	S	S	S	S
Aztreonam	S	S	S	S	S	S	S
Meropenem	S	S	S	S	S	S	S
Imipenem	S	S	S	S	S	S	S
Tetracycline	R	R	R	R	R	R	R

[a]Strains: 1, SZY H68; 2, SZY H8; 3, SZY H35; 4, SZY H36; 5, SZY H1^{T}; 6, *H. haemolyticus* NCTC 10659^{T}; 7, *H. influenzae* ATCC 10211. All data are from this study. S, sensitivity; I, intermediation; R, resistance.

TABLE S8 Detection of antibiotic resistance genes in the 23 *H. seminalis* strains[a].

	**1**	**2**	**3**	**4**	**5**	**6**	**7**	**8**	**9**	**10**	**11**	**12**	**13**	**14**	**15**	**16**	**17**	**18**	**19**	**20**	**21**	**22**	**23**	**Total**
CRP	+	+	+	+	+	+	–	+	+	+	+	+	+	–	–	+	+	+	+	+	+	+	+	20
TEM-1	–	–	–	–	–	–	–	+	–	–	–	–	–	–	–	–	–	–	–	–	–	–	–	1
hmrM	+	+	+	+	+	+	+	+	+	+	+	+	+	+	+	+	+	+	+	+	+	+	+	23
tet(B)	–	–	–	+	–	–	–	–	–	–	–	–	–	–	–	–	–	–	–	–	–	–	–	1
Total	2	2	2	3	2	2	1	3	2	2	2	2	2	1	1	2	2	2	2	2	2	2	2	45

[a]Strains are indicated as 1, SZY H1^{T}; 2, SZY H2; 3, H35; 4, H68; 5, H8; 6, H36; 7, C2001002324; 8, 60824 B Hi-4 ; 9, 65151 B Hi-4; 10, 60819 B Hi-1; 11, 60971 B Hi-3 ; 12, 60982 B Hi-1; 13, 839 HINF; 14, 65117 B Hi-3; 15, PN24; 16, BgEED18; 17, CCUG 11096; 18, CCUG 31732; 19, CCUG 30218; 20, CCUG 15949; 21, CCUG 66565; 22, Marseille-P9675; 23, F0629. –, not detected; +, detected.

FIG S1 Transmission electron micrographs of *H. seminalis* SZY H68 (A ,B) and *H. influenzae* ATCC 10211 (C, D) from cultures grown on *Haemophilus* chocolate 2 agar for 2 days under 35 °C and CO_2 (5%) atmosphere. A: The size bar was 1 μm. B, C and D: The size bar was 0.2 μm.

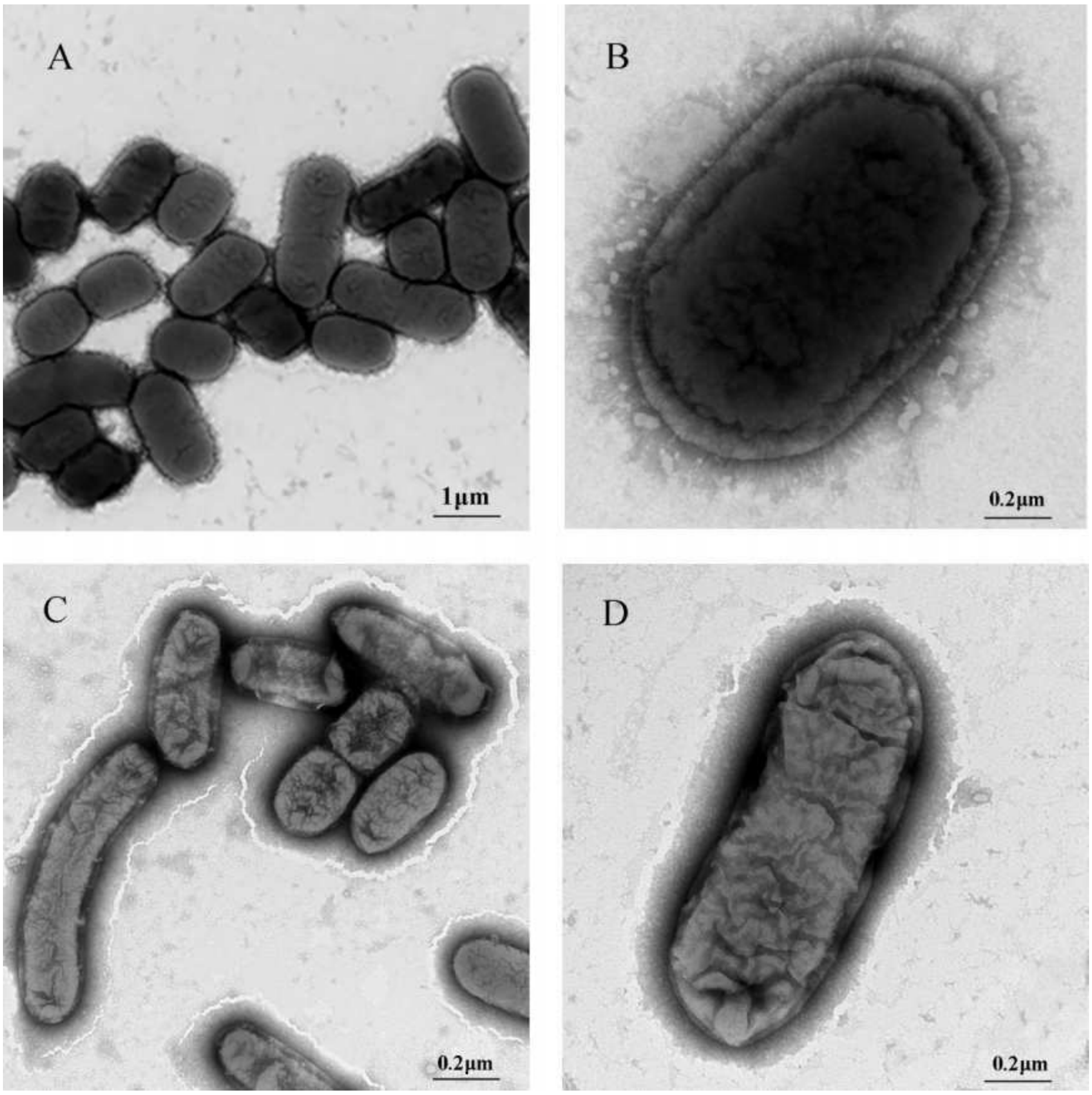

FIG S2 Heatmap of the average nucleotide identity (ANI) of 173 *Haemophilus* spp. strains. Percentage identity among strains is shown.

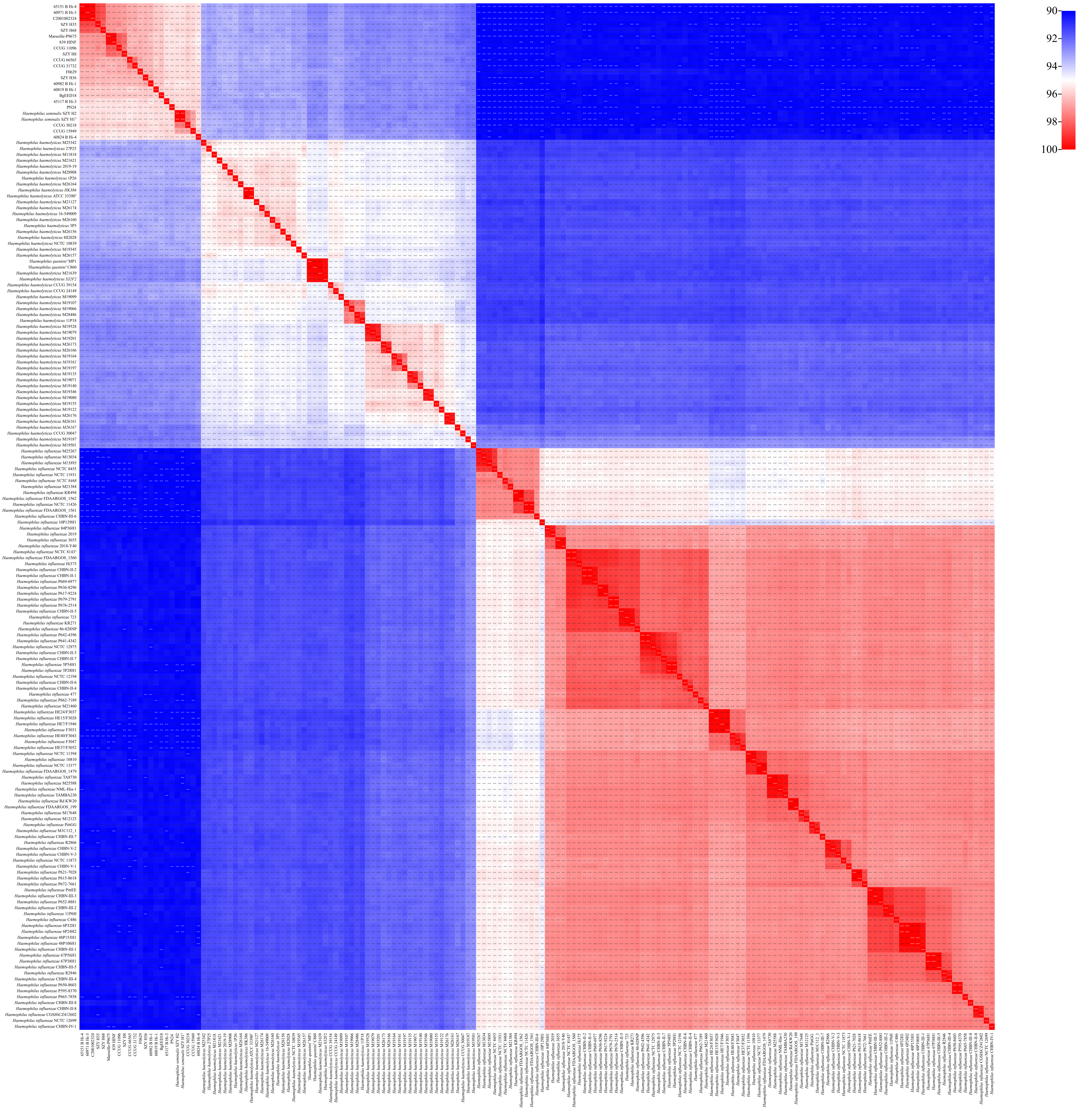

FIG S3 Phylogenetic trees of *Haemophilus* spp. strains. (A) Maximum likelihood tree based on partial 16S rRNA gene (about 996 bp) from 166 *Haemophilus* spp. strains. (B) Maximum likelihood tree based on partial 23S rRNA gene (about 1505 bp) from 169 *Haemophilus* spp. strains. (C) Maximum likelihood tree based on partial *rpoB* gene (about 1386 bp) from 173 *Haemophilus* spp. strains. (D) Maximum likelihood tree based on partial partial *recA* gene (about 1065 bp) from 172 *Haemophilus* spp. strains. (E) Maximum likelihood tree based on partial *frdB* gene (about 771 bp) from 173 *Haemophilus* spp. strains. (F) Maximum likelihood tree based on partial *mdh* gene (about 936 bp) from 171 *Haemophilus* spp. strains. (G) Maximum likelihood tree based on partial *adk* gene (about 645 bp) from 173 *Haemophilus* spp. strains. (H) Maximum likelihood tree based on partial *atpG* gene (about 645 bp) from 173 *Haemophilus* spp. strains. (I) Maximum likelihood tree based on partial *pgi* gene (about 854 bp) from 169 *Haemophilus* spp. strains. These single genes sequences were aligned using MUSCLE. The tree was made using IQ-TREE. Different colors of strain IDs stands for different species, as defined in the legend. The red strain ID represents *H. seminalis*, the blue strain ID represents *H. haemolyticus*, the green strain ID represents '*H. quentini*', the orange strain ID represents *H. influenzae*. The numbers at the nodes are support values estimated with 1000 bootstrap replicates. Only bootstrap values ≥90 are indicated.

FIG S3 (A) Maximum likelihood tree based on partial 16S rRNA gene (about 996 bp) from 166 *Haemophilus* spp. strains.

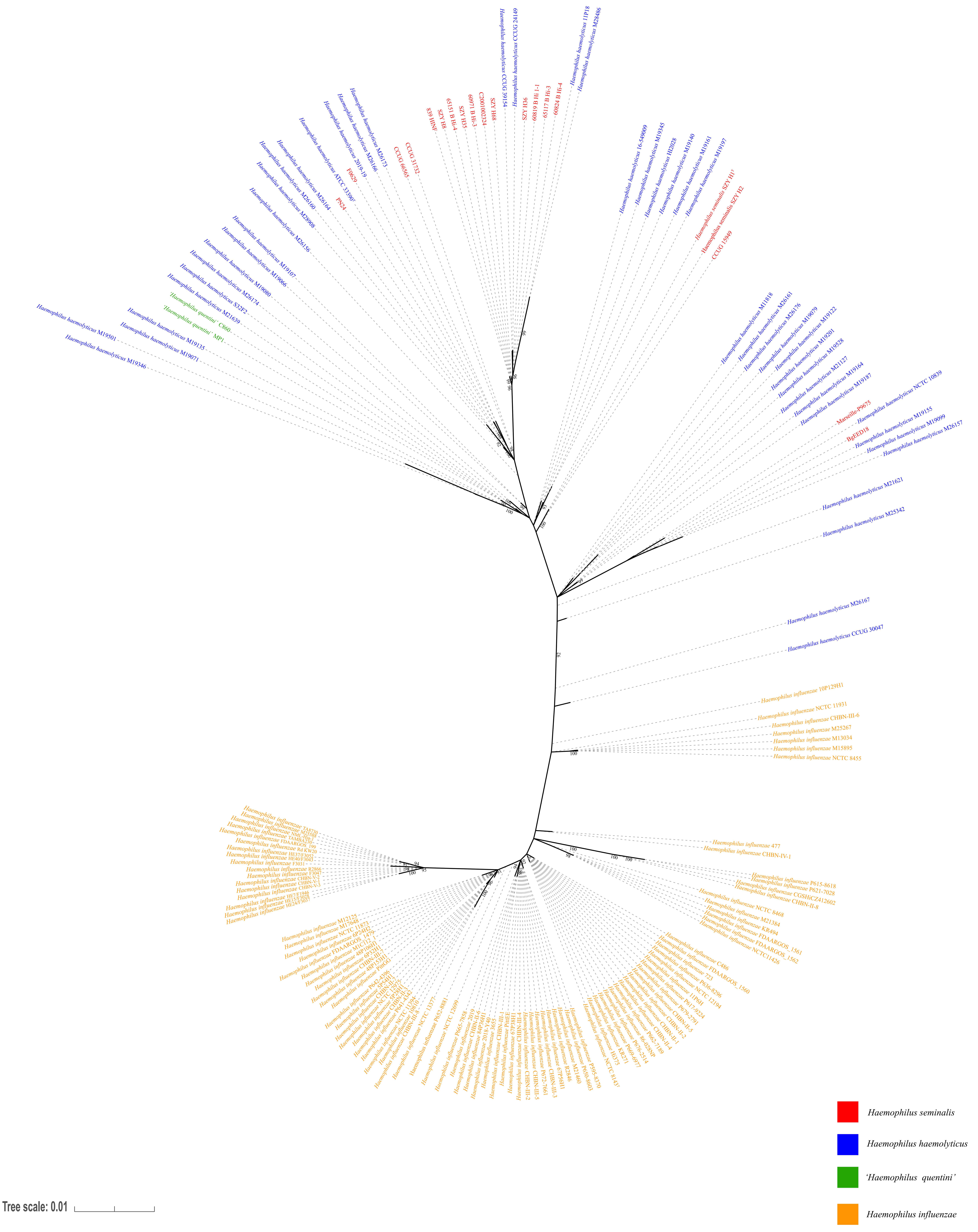

FIG S3 (B) Maximum likelihood tree based on partial 23S rRNA gene (about 1505 bp) from 169 *Haemophilus* spp. strains.

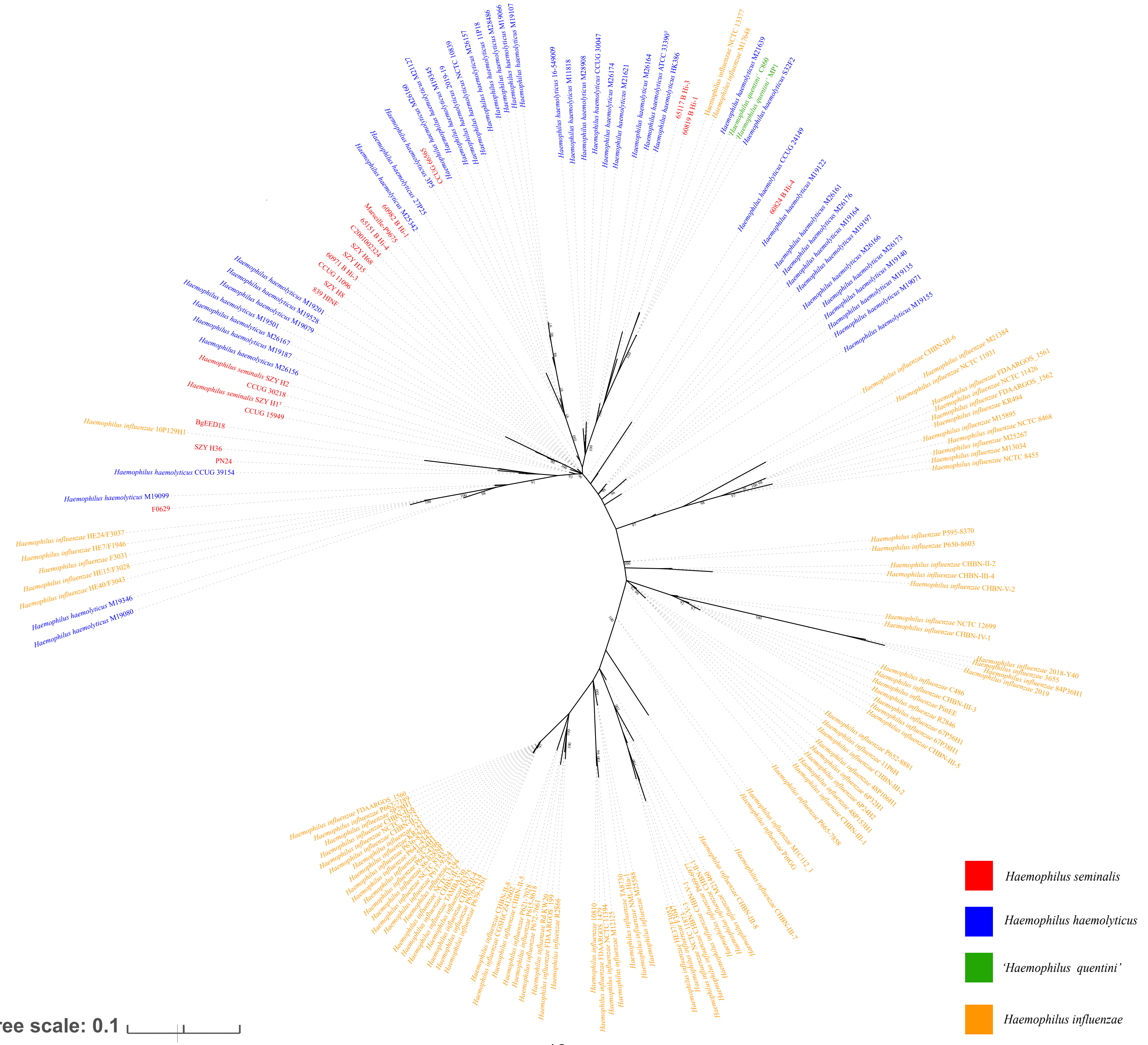

FIG S3 (C) Maximum likelihood tree based on partial *rpoB* gene (about 1386 bp) from 173 *Haemophilus* spp. strains.

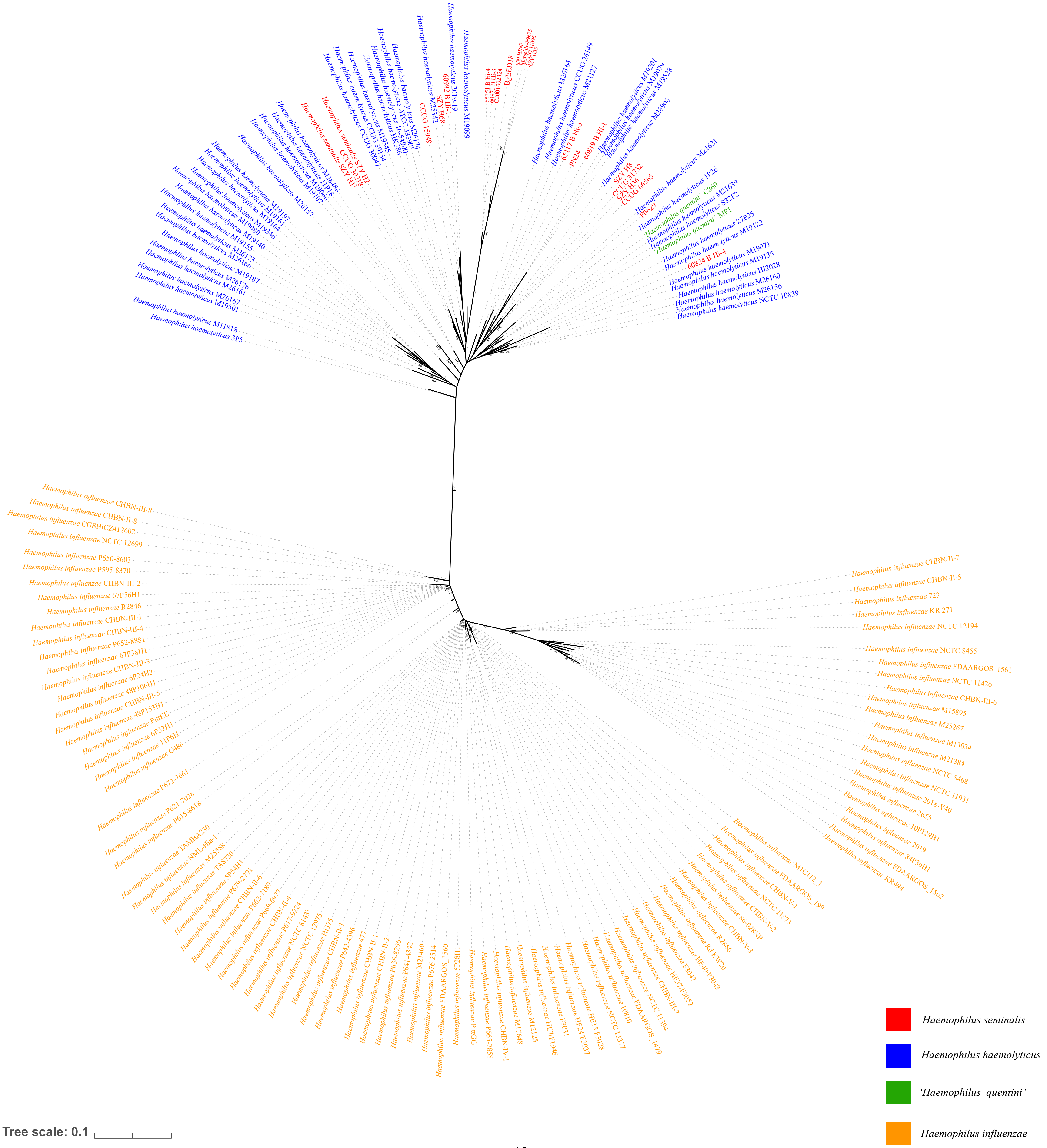

FIG S3 (D) Maximum likelihood tree based on partial *recA* gene (about 1065 bp) from 172 *Haemophilus* spp. strains.

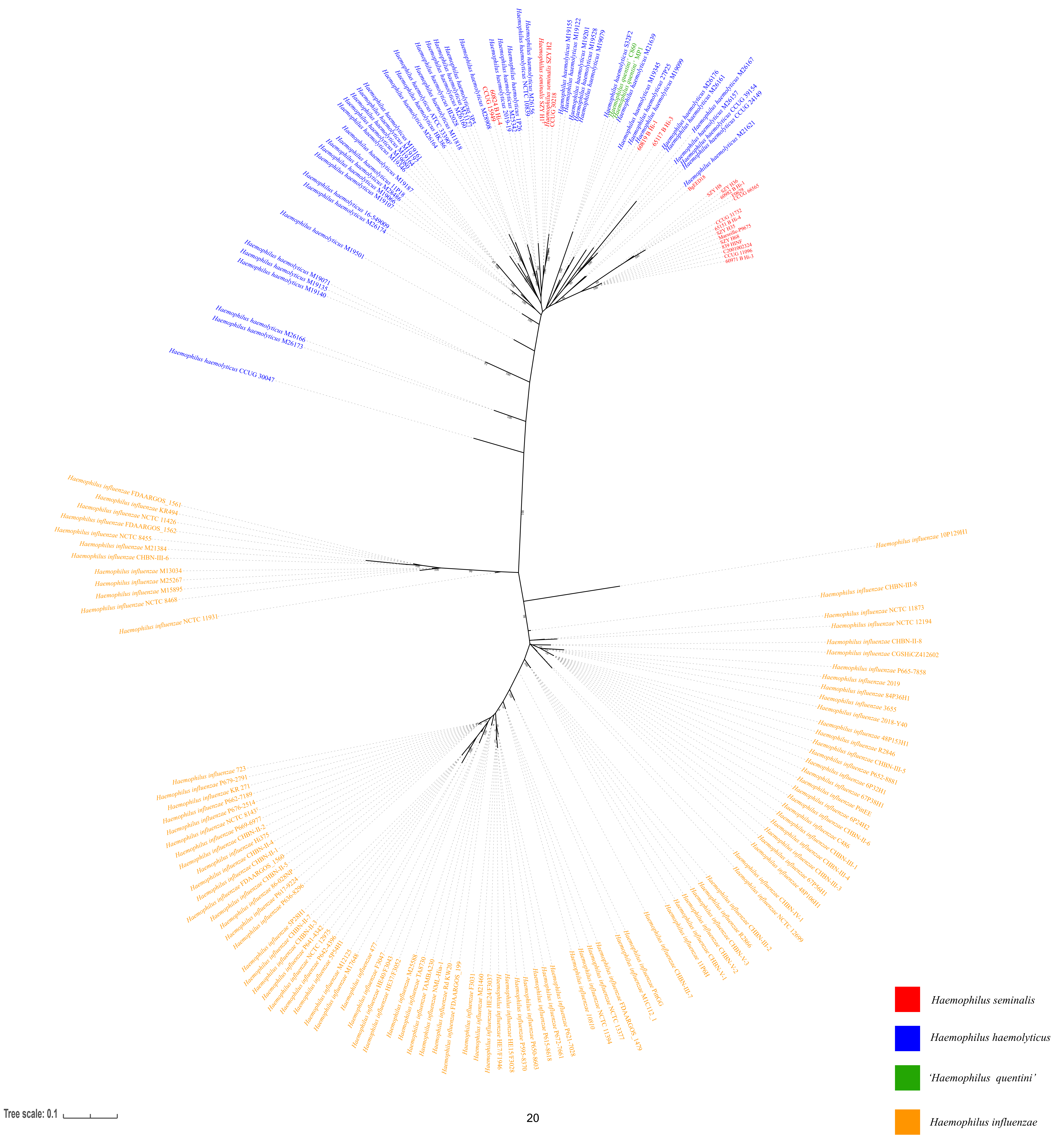

FIG S3 (E) Maximum likelihood tree based on partial *frdB* gene (about 771 bp) from 173 *Haemophilus* spp. strains.

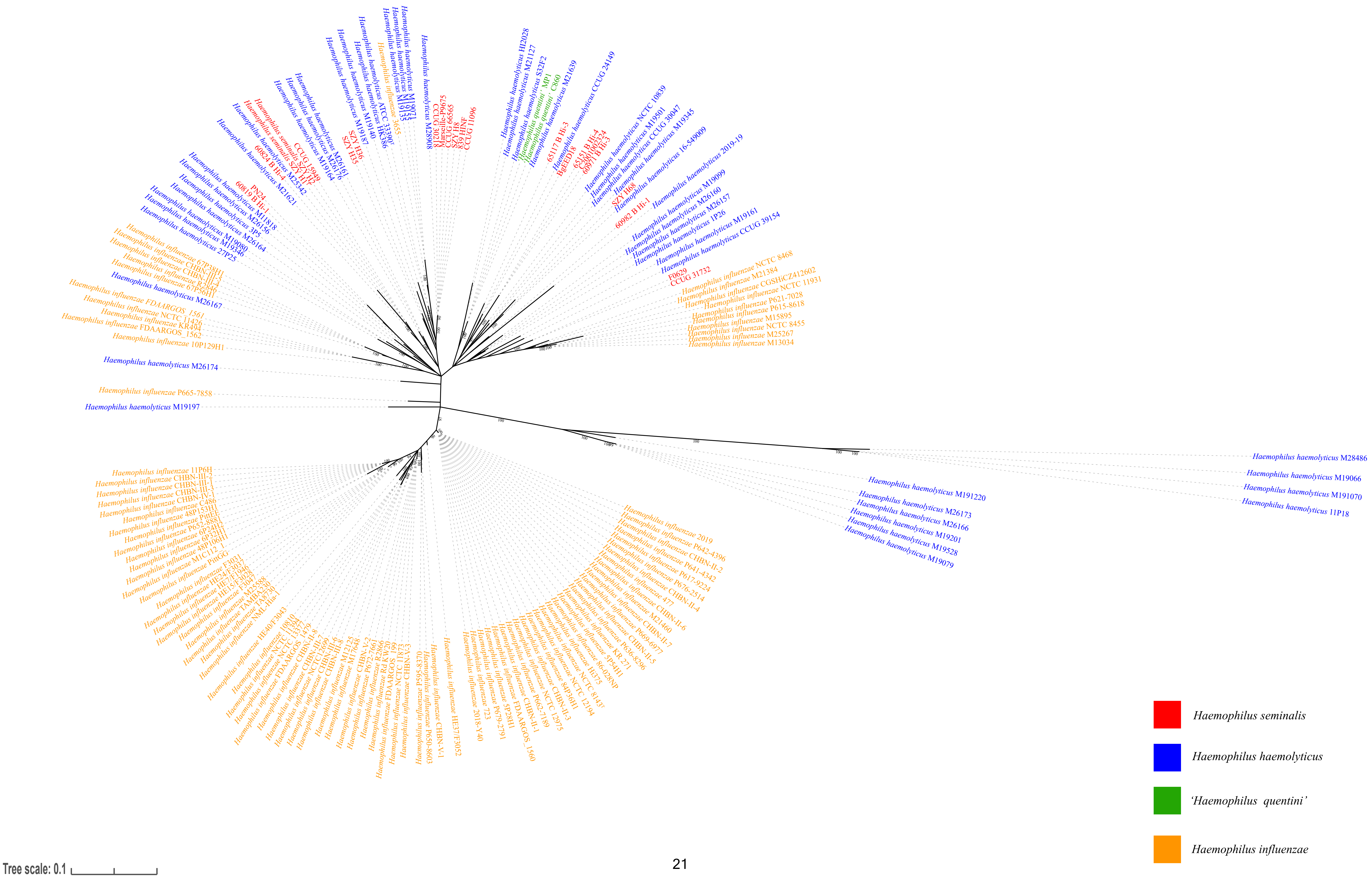

FIG S3 (F) Maximum likelihood tree based on partial *mdh* gene (about 936 bp) from 171 *Haemophilus* spp. strains.

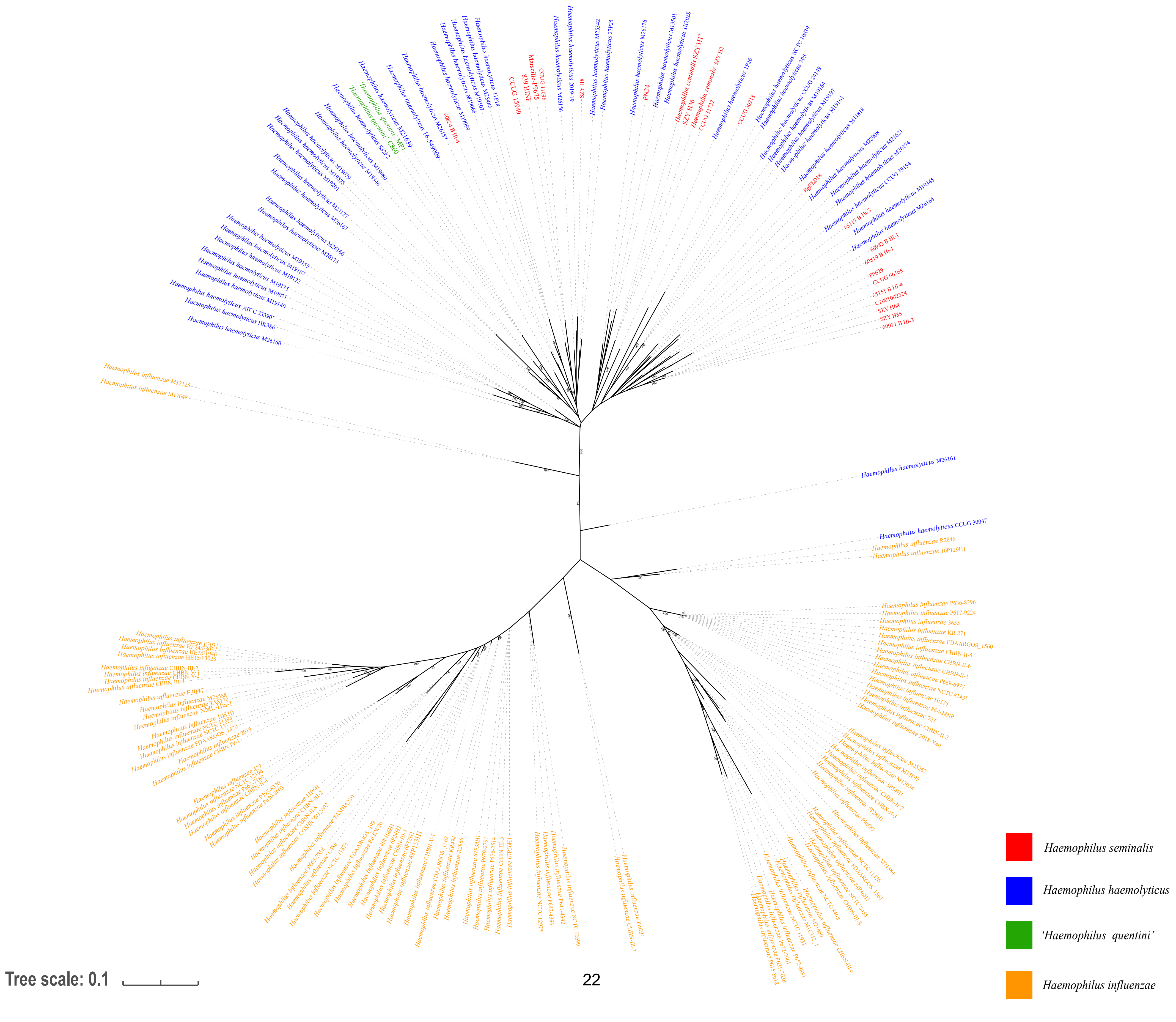

FIG S3 (G) Maximum likelihood tree based on partial *adk* gene (about 645 bp) from 173 *Haemophilus* spp. strains.

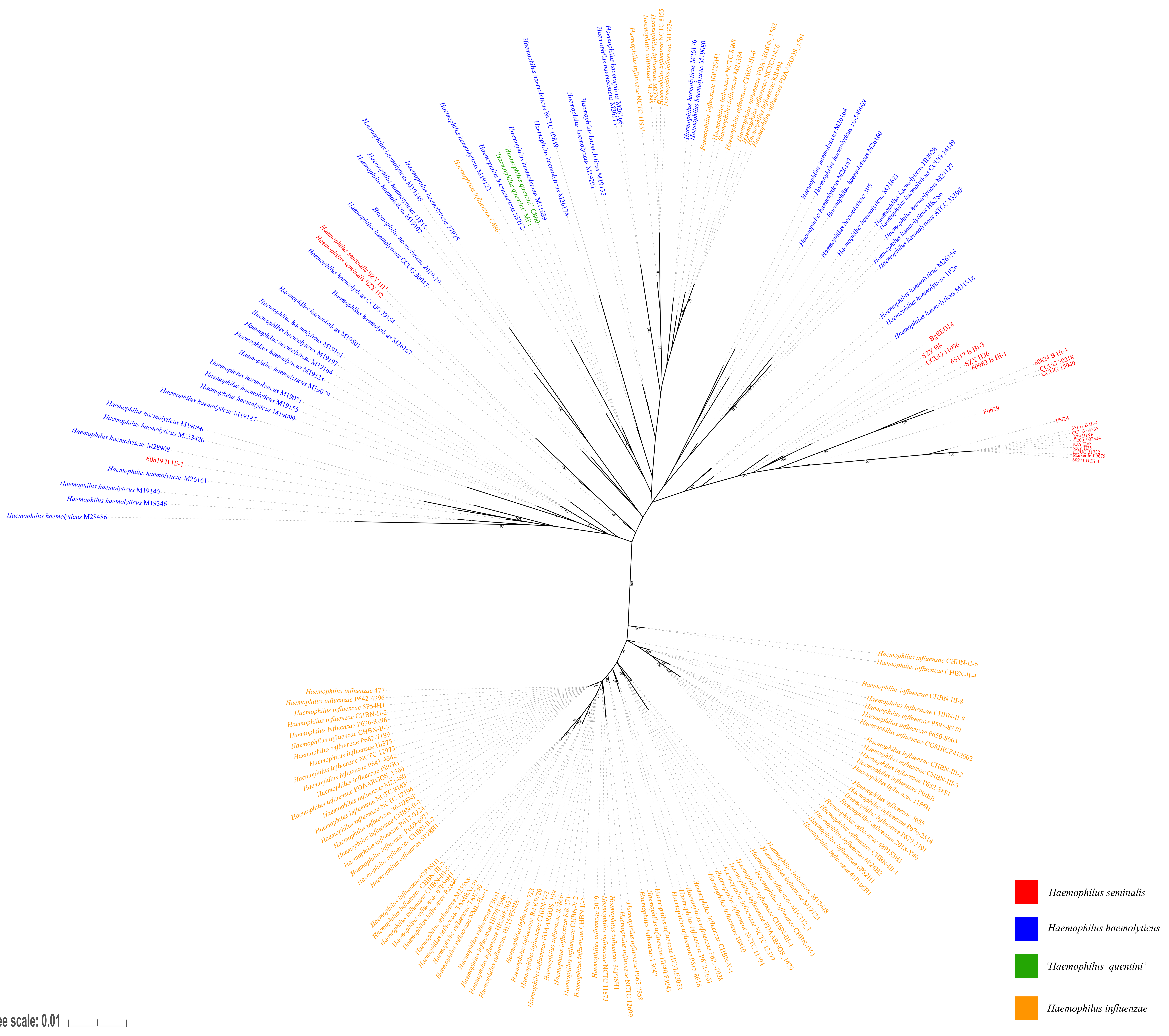

FIG S3 (H) Maximum likelihood tree based on partial *atpG* gene (about 645 bp) from 173 *Haemophilus* spp. strains.

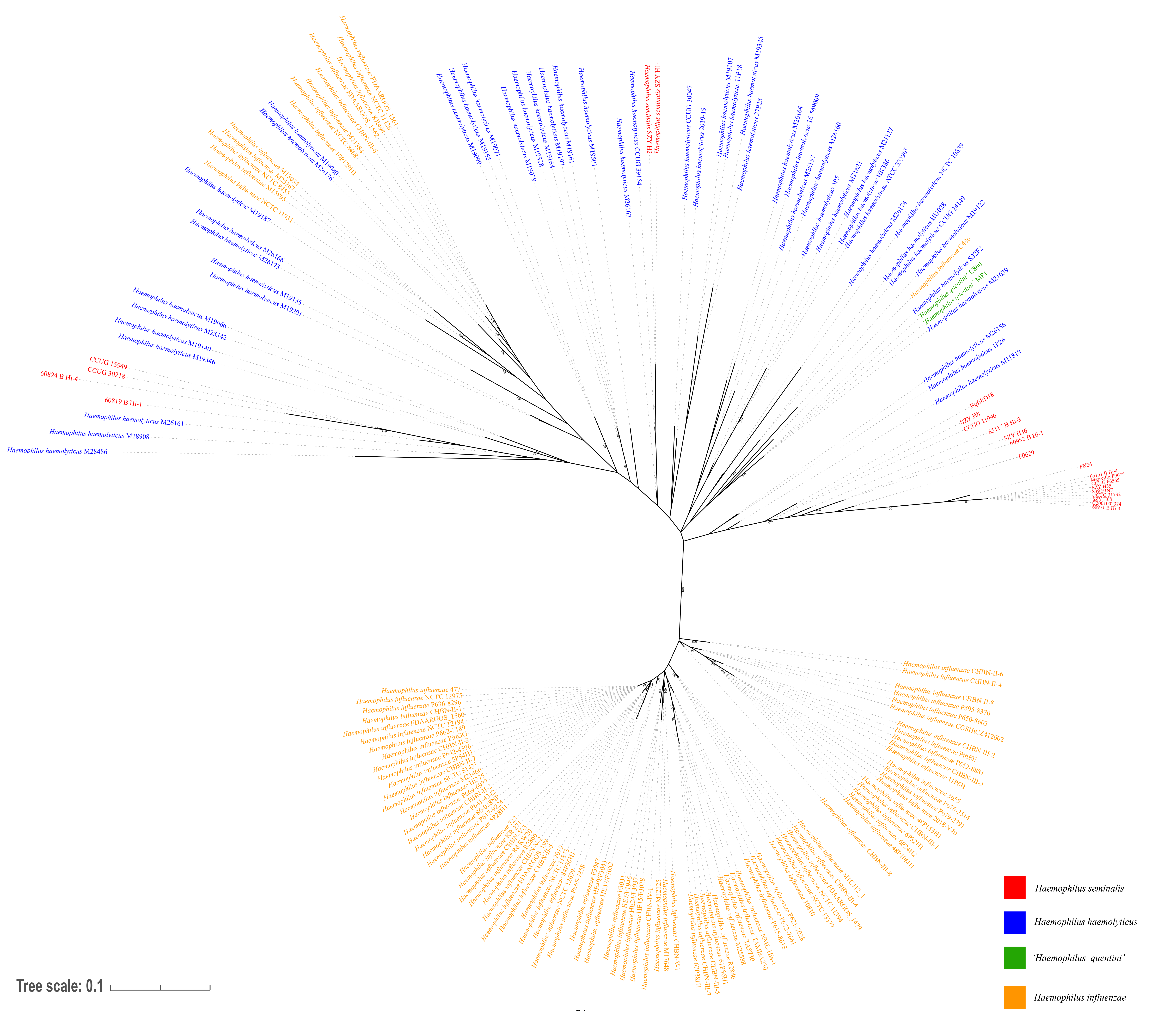

FIG S3 (I) Maximum likelihood tree based on partial *pgi* gene (about 854 bp) from 169 *Haemophilus* spp. strains.

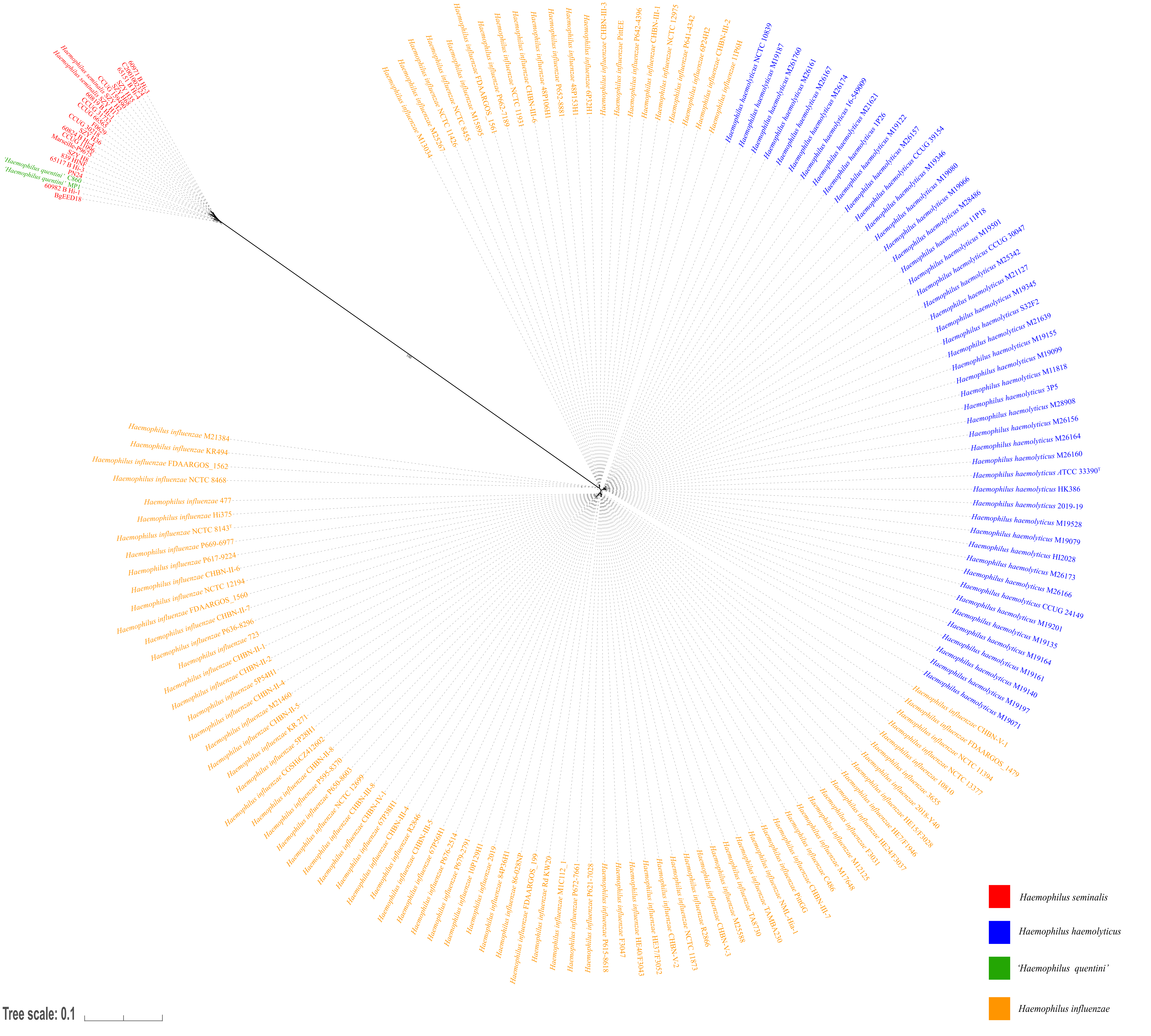

FIG S4 Phylogenetic trees of *Haemophilus* spp. strains. (A) Maximum likelihood tree based on partial *MoeA* gene (about 1212 bp) from 173 *Haemophilus* spp. strains. (B) Maximum likelihood tree based on partial *pepG* gene (about 1453 bp) from 173 *Haemophilus* spp. strains. These single genes sequences were aligned using MUSCLE. The tree was made using IQ-TREE. Different colors of strain IDs stands for different species, as defined in the legend. The red strain ID represents *H. seminalis*, the blue strain ID represents *H. haemolyticus*, the green strain ID represents '*H. quentini*', the orange strain ID represents *H. influenzae*. The numbers at the nodes are support values estimated with 1000 bootstrap replicates. Only bootstrap values ≥90 are indicated.

FIG S4 (A) Maximum likelihood tree based on partial *MoeA* gene (about 1212 bp) from 173 *Haemophilus* spp. strains.

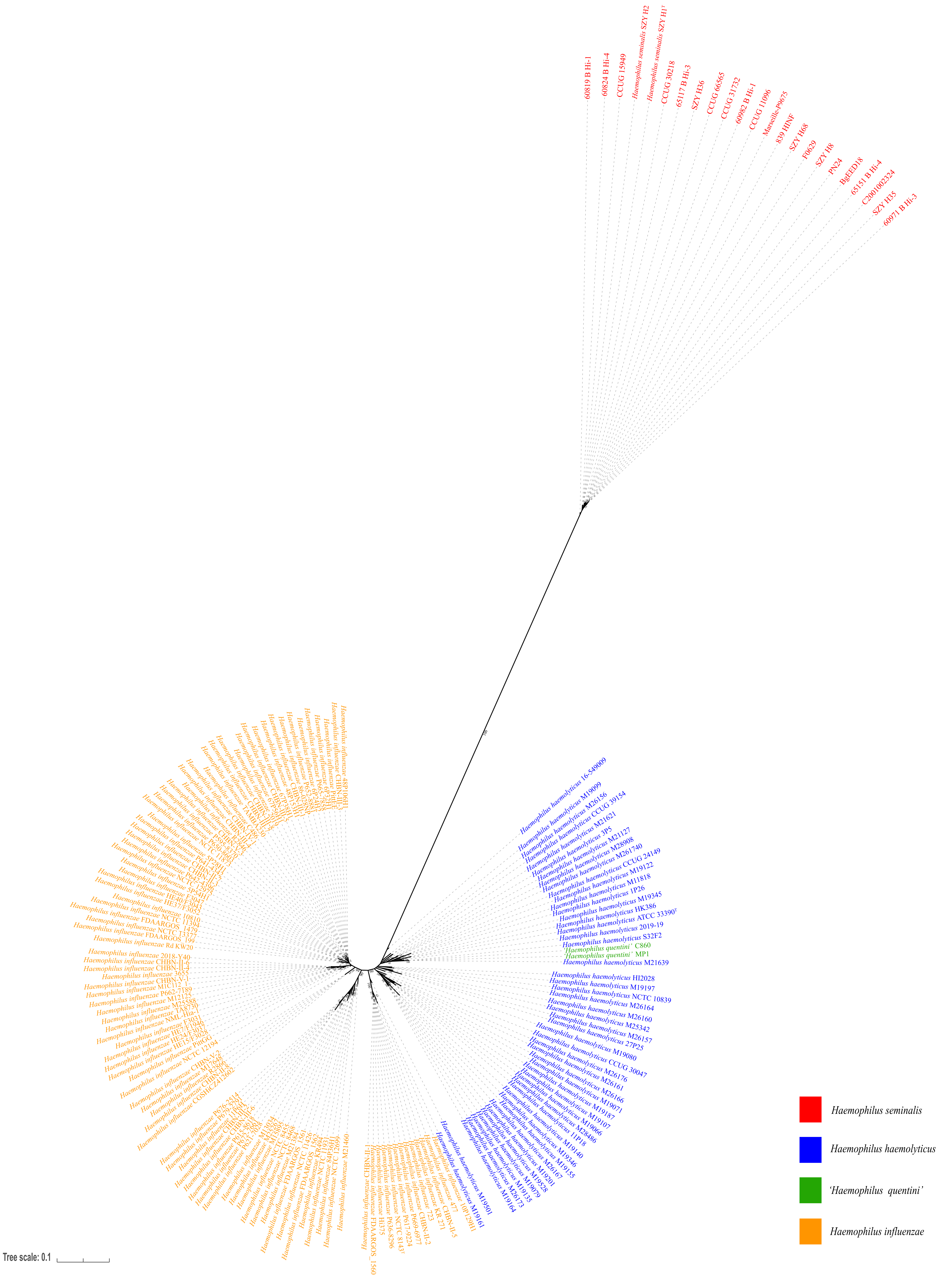

FIG S4 (B) Maximum likelihood tree based on partial *pepG* gene (about 1453 bp) from 173 *Haemophilus* spp. strains.

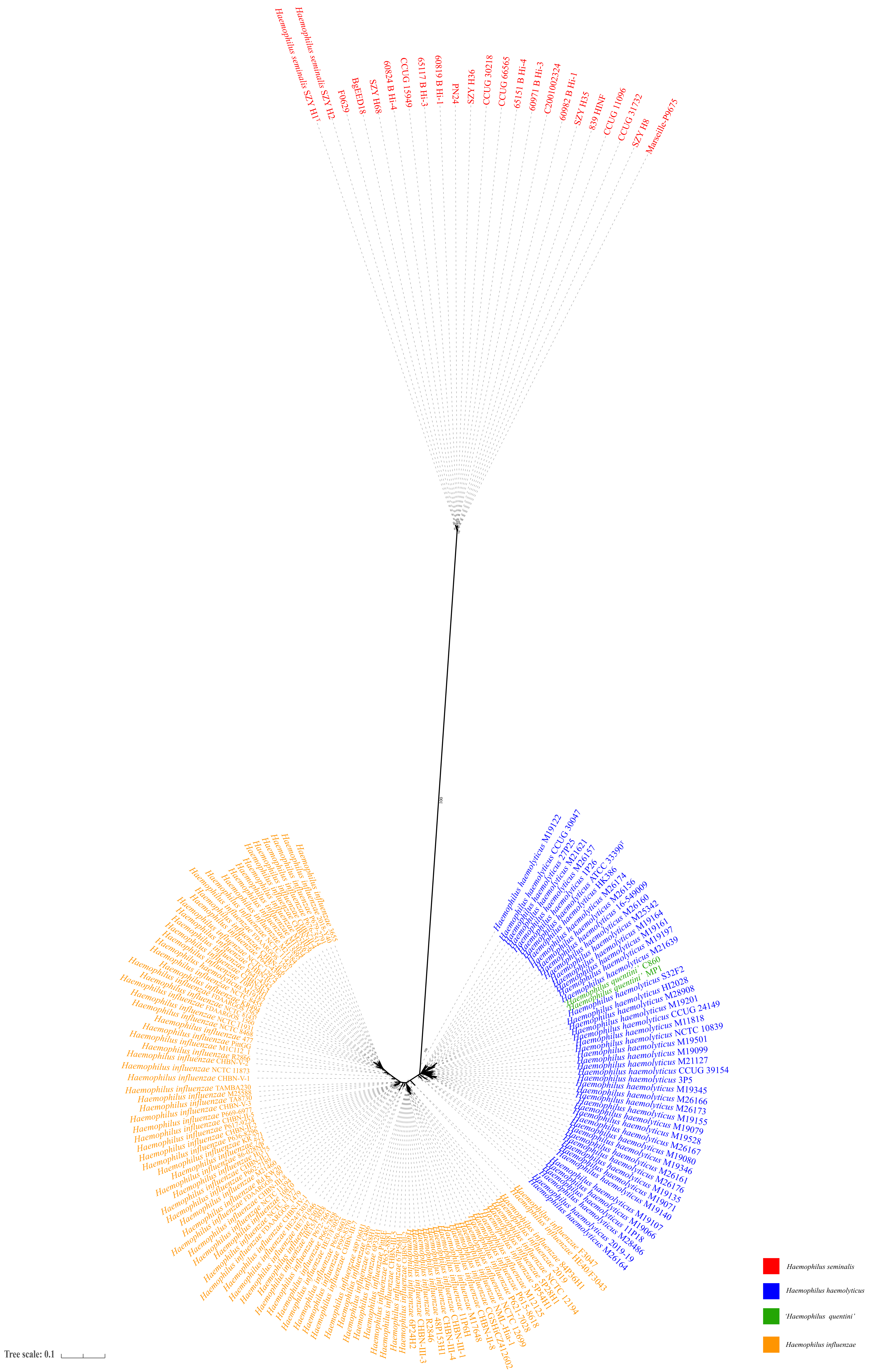

FIG S5 The COG functional annotations of 23 *H. seminalis* genomes. **(A)** Total number of genes annotated as Cluster of Orthologous Group (COG) from the pangenome. **(B)** COG annotation pangenome distribution. The COGs categories as A, RNA processing and modification; B, chromatin structure and dynamics; C, energy production and conversion; D, cell cycle control, cell division, chromosome partitioning; E, amino acid transport and metabolism; F, nucleotide transport and metabolism; G, carbohydrate transport and metabolism; H, coenzyme transport and metabolism; I, lipid transport and metabolism; translation; J, ribosomal structure and biogenesis ; K, transcription ; L, replication, recombination and repair ; M, cell wall/membrane/ envelope biogenesis; N, cell motility; O, post-translational modification, protein turnover, and chaperones; P, inorganic ion transport and metabolism; Q, secondary metabolites biosynthesis, transport and catabolism; S, function unknown; T, signal transduction mechanisms; U, intracellular trafficking, secretion, and vesicular transport; V, defense mechanisms; Z, cytoskeleton.

FIG S5 The COG functional annotations of 23 *H. seminalis* genomes. **(A)** Total number of genes annotated as Cluster of Orthologous Group (COG) from the pangenome. **(B)** COG annotation pangenome distribution.

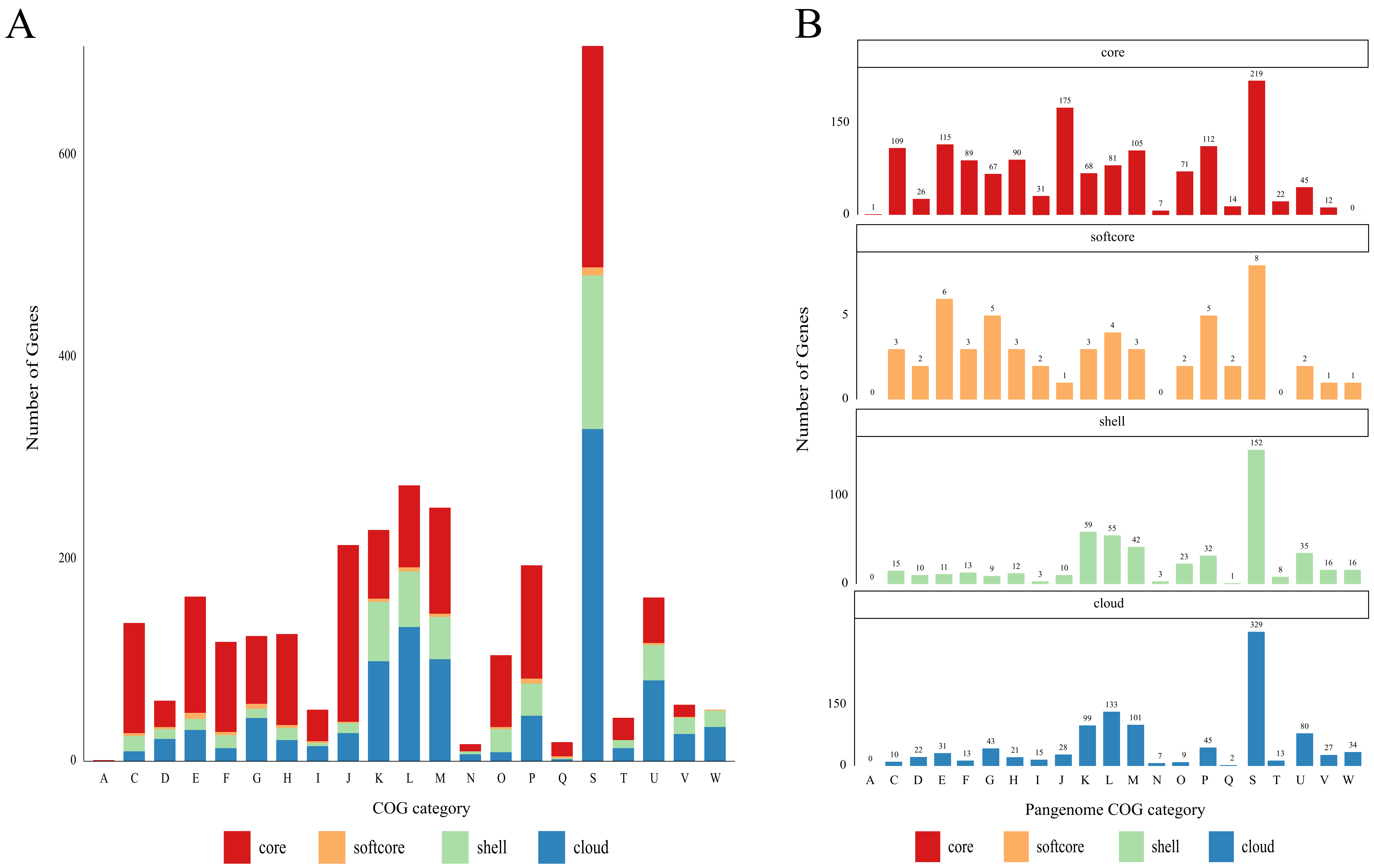

REFERENCE

1. Post DM, Ketterer MR, Coffin JE, Reinders LM, Munson RS Jr, Bair T, Murphy TF, Foster ED, Gibson BW, Apicella MA. 2016. Comparative analyses of the lipooligosaccharides from nontypeable *Haemophilus influenzae* and *Haemophilus haemolyticus* show differences in sialic acid and phosphorylcholine modifications. Infect Immun 84:765–774. https://doi.org/10.1128/IAI.01185-15.
2. Tanaka E, Hirai Y, Wajima T, Ishida Y, Kawamura Y, Nakaminami H. 2022. High-level quinolone-resistant *Haemophilus haemolyticus* in pediatric patient with no history of quinolone exposure. Emerg Infect Dis 28:104-110. https://doi.org/10.3201/eid2801.210248.
3. Topaz N, Boxrud D, Retchless AC, Nichols M, Chang HY, Hu F, Wang X. 2018. BMScan: using whole genome similarity to rapidly and accurately identify bacterial meningitis causing species. BMC Infect Dis 18:405. https://doi.org/10.1186/s12879-018-3324-1.
4. Jordan IK, Conley AB, Antonov IV, Arthur RA, Cook ED, Cooper GP, Jones BL, Knipe KM, Lee KJ, Liu X, Mitchell GJ, Pande PR, Petit RA, Qin S, Rajan VN, Sarda S, Sebastian A, Tang S, Thapliyal R, Varghese NJ, Ye T, Katz LS, Wang X, Rowe L, Frace M, Mayer LW. 2011. Genome sequences for five strains of the emerging pathogen *Haemophilus haemolyticus*. J Bacteriol 193:5879–5880. https://doi.org/10.1128/JB.05863-11.
5. Hubbard AT, Davies SE, Baxter L, Thompson S, Collery MM, Hand DC, Fink CG, Thomas DJ. 2016. Draft whole-genome sequence of a *Haemophilus* quentini strain isolated from an infant in the United Kingdom. Genome Announc 4:e01075-16. https://doi.org/10.1128/genomeA.01075-16.
6. Atack JM, Srikhanta YN, Fox KL, Jurcisek JA, Brockman KL, Clark TA, Boitano M, Power

PM, Jen FE, McEwan AG, Grimmond SM, Smith AL, Barenkamp SJ, Korlach J, Bakaletz LO, Jennings MP. 2015. A biphasic epigenetic switch controls immunoevasion, virulence and niche adaptation in non-typeable *Haemophilus influenzae*. Nat Commun 6:7828. https://doi.org/10.1038/ncomms8828.

7. Mell JC, Sinha S, Balashov S, Viadas C, Grassa CJ, Ehrlich GD, Nislow C, Redfield RJ, Garmendia J. 2014. Complete genome sequence of *Haemophilus influenzae* Strain 375 from the middle ear of a pediatric patient with otitis media. Genome Announc 2:e01245-14. https://doi.org/10.1128/genomeA.01245-14.
8. Moleres J, Fernández-Calvet A, Ehrlich RL, Martí S, Pérez-Regidor L, Euba B, Rodríguez-Arce I, Balashov S, Cuevas E, Liñares J, Ardanuy C, Martín-Santamaría S, Ehrlich GD, Mell JC, Garmendia J. 2018. Antagonistic pleiotropy in the bifunctional surface protein fadl (ompp1) during adaptation of *Haemophilus influenzae* to chronic lung infection associated with chronic obstructive pulmonary disease. mBio 9:e01176-18. https://doi.org/10.1128/mBio.01176-18.
9. Tanaka E, Wajima T, Nakaminami H, Noguchi N. 2020. Whole-genome sequence of *Haemophilus influenzae* ST422 outbreak clone strain 2018-Y40 with low quinolone susceptibility isolated from a paediatric patient. J Glob Antimicrob Resist 22:759–761. https://doi.org/10.1016/j.jgar.2020.06.024.
10. Kubota M, Kenri T, Sasaki Y, Shibayama K, Takayanagi K, Kenzaka T. 2020. Complete genome sequence of a Japanese clinical isolate of *Haemophilus influenzae* type a strain TAMBA230. Microbiol Resour Announc 9:e01069-20. https://doi.org/10.1128/MRA.01069-20.

11. Watts SC, Judd LM, Carzino R, Ranganathan S, Holt KE. 2021. Genomic diversity and antimicrobial resistance of *Haemophilus* colonizing the airways of young children with cystic fibrosis. mSystems 31:e0017821. https://doi.org/10.1128/mSystems.00178-21.

12. Harrison A, Dyer DW, Gillaspy A, Ray WC, Mungur R, Carson MB, Zhong H, Gipson J, Gipson M, Johnson LS, Lewis L, Bakaletz LO, Munson RS Jr. 2005. Genomic sequence of an otitis media isolate of nontypeable *Haemophilus influenzae*: comparative study with *H. influenzae* serotype d, strain KW20. J Bacteriol 187:4627–4636. https://doi.org/10.1128/JB.187.13.4627–4636.2005.

13. Strouts FR, Power P, Croucher NJ, Corton N, van Tonder A, Quail MA, Langford PR, Hudson MJ, Parkhill J, Kroll JS, Bentley SD. 2012. Lineage-specific virulence determinants of *Haemophilus influenzae* biogroup aegyptius. Emerg Infect Dis 18:449–457. https://doi.org/10.3201/eid1803.110728.

14. Su YC, Hörhold F, Singh B, Riesbeck K. 2013. Complete genome sequence of encapsulated *Haemophilus influenzae* Type f KR494, an invasive isolate that caused necrotizing myositis. Genome Announc 1:e00470-13. https://doi.org/10.1128/genomeA.00470-13.

15. Hogg JS, Hu FZ, Janto B, Boissy R, Hayes J, Keefe R, Post JC, Ehrlich GD. 2007. Characterization and modeling of the *Haemophilus influenzae* core and supragenomes based on the complete genomic sequences of Rd and 12 clinical nontypeable strains. Genome Biol 8:R103. https://doi.org/10.1186/gb-2007-8-6-r103.

16. Erwin AL, Nelson KL, Mhlanga-Mutangadura T, Bonthuis PJ, Geelhood JL, Morlin G, Unrath WC, Campos J, Crook DW, Farley MM, Henderson FW, Jacobs RF, Mühlemann K, Satola SW, van Alphen L, Golomb M, Smith AL. 2005. Characterization of genetic and

phenotypic diversity of invasive nontypeable *Haemophilus influenzae*. Infect Immun 73:5853–5863. https://doi.org/ 10.1128/IAI.73.9.5853-5863.2005.

17. Fleischmann RD, Adams MD, White O, Clayton RA, Kirkness EF, Kerlavage AR, Bult CJ, Tomb JF, Dougherty BA, Merrick JM, McKenney K, Sutton G , FitzHugh W, Fields C, Gocayne JD, Scott J, Shirley R, Liu L, Glodek A, Kelley JM, Weidman JF, Phillips CA, Spriggs T, Hedblom E, Cotton MD, Utterback TR, Hanna MC, Nguyen DT, Saudek DM, Brandon RC, Fine LD, Fritchman JL, Fuhrmann JL, Geoghagen N, Gnehm CL, McDonald LA, Small KV, Fraser CM, Smith HO, Venter JC. 1995. Whole-genome random sequencing and assembly of *Haemophilus influenzae* Rd. Science 269:496–512. https://doi.org/10.1126/science.7542800.
